# Assessment and economic valuation of air pollution impacts on human health over Europe and the United States as calculated by a multi-model ensemble in the framework of AQMEII3

**DOI:** 10.5194/acp-18-5967-2018

**Published:** 2018-04-27

**Authors:** Ulas Im, Jørgen Brandt, Camilla Geels, Kaj Mantzius Hansen, Jesper Heile Christensen, Mikael Skou Andersen, Efisio Solazzo, Ioannis Kioutsioukis, Ummugulsum Alyuz, Alessandra Balzarini, Rocio Baro, Roberto Bellasio, Roberto Bianconi, Johannes Bieser, Augustin Colette, Gabriele Curci, Aidan Farrow, Johannes Flemming, Andrea Fraser, Pedro Jimenez-Guerrero, Nutthida Kitwiroon, Ciao-Kai Liang, Uarporn Nopmongcol, Guido Pirovano, Luca Pozzoli, Marje Prank, Rebecca Rose, Ranjeet Sokhi, Paolo Tuccella, Alper Unal, Marta Garcia Vivanco, Jason West, Greg Yarwood, Christian Hogrefe, Stefano Galmarini

**Affiliations:** 1 Aarhus University, Department of Environmental Science, Frederiksborgvej 399, Roskilde, Denmark; 2 European Commission, Joint Research Centre (JRC), Ispra, Italy; 3 University of Patras, Department of Physics, University Campus 26504 Rio, Patras, Greece; 4 Eurasia Institute of Earth Sciences, Istanbul Technical University, Istanbul, Turkey; 5 Ricerca sul Sistema Energetico (RSE S.p.A.), Milan, Italy; 6 University of Murcia, Department of Physics, Physics of the Earth, Campus de Espinardo, Ed. CIOyN, Murcia, Spain; 7 Enviroware SRL, Concorezzo MB, Italy; 8 Institute of Coastal Research, Chemistry Transport Modelling Group, Helmholtz-Zentrum Geesthacht, Geesthacht, Germany; 9 INERIS, Institut National de l’Environnement Industriel et des Risques, Parc Alata, Verneuil-en-Halatte, France; 10 Dept. Physical and Chemical Sciences, University of L’Aquila, L’Aquila, Italy; 11 Center of Excellence CETEMPS, University of L’Aquila, L’Aquila, Italy; 12 Centre for Atmospheric and Instrumentation Research (CAIR), University of Hertfordshire, Hatfield, UK; 13 European Centre for Medium Range Weather Forecast (ECMWF), Reading, UK; 14 Ricardo Energy & Environment, Gemini Building, Fermi Avenue, Harwell, Oxon, UK; 15 Environmental Research Group, Kings’ College London, London, UK; 16 Department of Environmental Sciences and Engineering, University of North Carolina at Chapel Hill, Chapel Hill, NC, USA; 17 Ramboll Environ, 773 San Marin Drive, Suite 2115, Novato, CA, USA; 18 Finnish Meteorological Institute, Atmospheric Composition Research Unit, Helsinki, Finland; 19 Cornell University, Department of Earth and Atmospheric Sciences, Ithaca, NY, USA; 20 CIEMAT. Avda. Complutense 40., Madrid, Spain; 21 Computational Exposure Division, National Exposure Research Laboratory, Office of Research and Development, United States Environmental Protection Agency, Research Triangle Park, NC, USA

## Abstract

The impact of air pollution on human health and the associated external costs in Europe and the United States (US) for the year 2010 are modeled by a multi-model ensemble of regional models in the frame of the third phase of the Air Quality Modelling Evaluation International Initiative (AQMEII3). The modeled surface concentrations of O_3_, CO, SO_2_ and PM_2.5_ are used as input to the Economic Valuation of Air Pollution (EVA) system to calculate the resulting health impacts and the associated external costs from each individual model. Along with a base case simulation, additional runs were performed introducing 20 % anthropogenic emission reductions both globally and regionally in Europe, North America and east Asia, as defined by the second phase of the Task Force on Hemispheric Transport of Air Pollution (TF-HTAP2).

Health impacts estimated by using concentration inputs from different chemistry–transport models (CTMs) to the EVA system can vary up to a factor of 3 in Europe (12 models) and the United States (3 models). In Europe, the multi-model mean total number of premature deaths (acute and chronic) is calculated to be 414 000, while in the US, it is estimated to be 160 000, in agreement with previous global and regional studies. The economic valuation of these health impacts is calculated to be EUR 300 billion and 145 billion in Europe and the US, respectively. A subset of models that produce the smallest error compared to the surface observations at each time step against an all-model mean ensemble results in increase of health impacts by up to 30 % in Europe, while in the US, the optimal ensemble mean led to a decrease in the calculated health impacts by ~ 11 %.

A total of 54 000 and 27 500 premature deaths can be avoided by a 20 % reduction of global anthropogenic emissions in Europe and the US, respectively. A 20 % reduction of North American anthropogenic emissions avoids a total of ~ 1000 premature deaths in Europe and 25 000 total premature deaths in the US. A 20 % decrease of anthropogenic emissions within the European source region avoids a total of 47 000 premature deaths in Europe. Reducing the east Asian anthropogenic emissions by 20 % avoids ~ 2000 total premature deaths in the US. These results show that the domestic anthropogenic emissions make the largest impacts on premature deaths on a continental scale, while foreign sources make a minor contribution to adverse impacts of air pollution.

## 1 Introduction

According to the World Health Organization (WHO), air pollution is now the world’s largest single environmental health risk (WHO, 2014). Around 7 million people died prematurely in 2012 as a result of air pollution exposure from both outdoor and indoor emission sources (WHO, 2014). WHO estimates 3.7 million premature deaths in 2012 from exposure to outdoor air pollution from urban and rural sources worldwide. According to the Global Burden of Disease (GBD) study, exposure to ambient particulate matter pollution remains among the 10 leading risk factors. Air pollution is a transboundary phenomenon with global, regional, national and local sources, leading to large differences in the geographical distribution of human exposure. Short-term exposure to ozone (O_3_) is associated with respiratory morbidity and mortality (e.g., Bell et al., 2004), while long-term exposure to O_3_ has been associated with premature respiratory mortality (Jerrett et al., 2009). Short-term exposure to particulate matter (PM_2.5_) has been associated with increases in daily mortality rates from respiratory and cardiovascular causes (e.g., Pope and Dockery, 2006), while long-term exposure to PM_2.5_ can have detrimental chronic health effects, including premature mortality due to cardiopulmonary diseases and lung cancer (Burnett et al., 2014). The Global Burden of Disease Study 2015 estimated 254 000 O_3_-related and 4.2 million anthropogenic PM_2.5_-related premature deaths per year (Cohen et al., 2017).

Changes in emissions from one region can impact air quality over others, affecting also air-pollution-related health impacts due to intercontinental transport (Anenberg et al., 2014; Zhang et al., 2017). In the framework of the Task Force on Hemispheric Transport of Air Pollution (TF-HTAP), Anenberg et al. (2009) found that reduction of foreign ozone precursor emissions can contribute to more than 50 % of the deaths avoided by simultaneously reducing both domestic and foreign precursor emissions. Similarly, they found that reducing emissions in North America (NA) and Europe (EU) has the largest impacts on ozone-related premature deaths in downwind regions than within (Anenberg et al., 2009). This result agrees with Duncan et al. (2008), who showed for the first time that emission reductions in NA and EU have greater impacts on ozone mortality outside the source region than within. Anenberg et al. (2014) estimates that 93–97 % of PM_2.5_-related avoided deaths from reducing emissions occur within the source region while 3–7 % occur outside the source region from concentrations transported between continents. In spite of the shorter lifetime of PM_2.5_ compared to O_3_, it was found to cause more deaths from intercontinental transport (Anenberg et al., 2009, 2014). In the frame of the second phase of the Task Force on Hemispheric Transport of Air Pollution (TF-HTAP2; Galmarini et al., 2017), an ensemble of global chemistry–transport model simulations calculated that 20 % emission reductions from one region generally lead to more avoided deaths within the source region than outside (Liang et al., 2018).

Recently, Lelieveld et al. (2015) used a global chemistry model and calculated that outdoor air pollution led to 3.3 million premature deaths globally in 2010. They calculated that, in Europe and North America, 381 000 and 68 000 premature deaths occurred, respectively. They have also calculated that these numbers are likely to roughly double in the year 2050 assuming a business-as-usual scenario. Silva et al. (2016), using the Atmospheric Chemistry and Climate Model Intercomparison Project (ACCMIP) model ensemble, calculated that the global mortality burden of ozone is estimated to markedly increase from 382 000 deaths in 2000 to between 1.09 and 2.36 million in 2100. They also calculated that the global mortality burden of PM_2.5_ is estimated to decrease from 1.70 million deaths in 2000 to between 0.95 and 1.55 million deaths in 2100. Silva et al. (2013) estimated that in 2000, 470 000 premature respiratory deaths are associated globally and annually with anthropogenic ozone and 2.1 million deaths with anthropogenic PM_2.5_-related cardiopulmonary diseases (93 %) and lung cancer (7 %). These studies employed global chemistry–transport models with coarse spatial resolution (≥ 0.5° × 0.5°); therefore, health benefits from reducing local emissions were not able to be adequately captured. Higher resolutions are necessary to calculate more robust estimates of health benefits from local vs. non-local sources (Fenech et al., 2017). In addition, these studies calculated the number of premature deaths due to air pollution; however, none of them address morbidity such as number of lung cancer or asthma cases, or restricted activity days. Finally, these studies did not include economic costs either. On the other hand, there are a number of regional studies that calculate health impacts on finer spatial resolutions and address morbidity. However, they are mostly based on single air pollution models or do not evaluate the health benefits from local vs. non-local emissions. Therefore, a comprehensive study employing a multi-model ensemble of high spatial resolution and focusing on both mortality and morbidity from local vs. non-local sources is lacking in the literature.

In Europe, recent results show that outdoor air pollution due to O_3_, CO, SO_2_ and PM_2.5_ causes a total number of 570 000 premature deaths in the year 2011 (Brandt et al., 2013a, b). The external (or indirect) costs to society related to health impacts from air pollution are tremendous. OECD (2014) estimates that outdoor air pollution is costing its member countries USD 1.57 trillion in 2010. Among the OECD member countries, the economic valuation of air pollution in the US was calculated to be ~ USD 500 billion, and ~ USD 660 billion in Europe. In all of Europe, the total external costs have been estimated to approximately EUR 800 billion in the year 2011 (Brandt et al., 2013a). These societal costs have great influence on the general level of welfare and especially on the distribution of welfare both within the countries, as air pollution levels are vastly heterogeneous both at regional and local scales, and between the countries, as air pollution and the related health impacts are subject to long-range transport. Geels et al. (2015), using two regional chemistry–transport models, estimated a premature mortality of 455 000 and 320 000 in the 28 member states of the European Union (EU-28) for the year 2000, respectively, due to O_3_, CO, SO_2_ and PM_2.5_. They also estimated that climate change alone will lead to a small increase (15 %) in the total number of O_3_-related acute premature deaths in Europe towards the 2080s and relatively small changes (< 5 %) for PM_2.5_-related mortality. They found that the combined effect of climate change and emission reductions will reduce the premature mortality due to air pollution, in agreement with the results from Schucht et al. (2015).

The US Environmental Protection Agency estimated that in 2010 there were ~ 160 000 premature deaths in the US due to air pollution (US EPA, 2011). Fann et al. (2012) calculated 130 000–350 000 premature deaths associated with O_3_ and PM_2.5_ from the anthropogenic sources in the US for the year 2005. Caiazzo et al. (2013) estimated 200 000 cases of premature deaths in the US due to air pollution from combustion sources for the year 2005.

The health impacts of air pollution and their economic valuation are estimated based on observed and/or modeled air pollutant concentrations. Observations have spatial limitations particularly when assessments are needed for large regions. The impacts of air pollution on health can be estimated using models, where the level of complexity can vary depending on the geographical scale (global, continental, country or city), concentration input (observations, model calculations, emissions) and the pollutants of interest that can vary from only few (PM_2.5_ or O_3_) to a whole set of all regulated pollutants. The health impact models normally used may differ in the geographical coverage, spatial resolutions of the air pollution model applied, complexity of described processes, the exposure–response functions (ERFs), population distributions and the baseline indices (see Anenberg et al., 2015 for a review).

Air-pollution-related health impacts and associated costs can be calculated using a chemistry–transport model (CTM) or with standardized source–receptor relationships characterizing the dependence of ambient concentrations on emissions (e.g., EcoSense model: ExternE, 2005; TM5-FASST: Van Dingenen et al., 2014). Source–receptor relationships have the advantage of reducing the computing time significantly and have therefore been extensively used in systems like GAINS (Amann et al., 2011). On the other hand, full CTM simulations have the advantage of better accounting for non-linear chemistry–transport processes in the atmosphere.

CTMs are useful tools to calculate the concentrations of health-related pollutants taking into account non-linearities in the chemistry and the complex interactions between meteorology and chemistry. However, the CTMs include different chemical and aerosol schemes that introduce differences in the representation of the atmosphere as well as differences in the emissions and boundary conditions they use (Im et al., 2015a, b). These different approaches are present also in the health impact estimates that use CTM results as the basis for their calculations. Multi-model (MM) ensembles can be useful to the extent that allows us to take into consideration several model results at the same time, define the relative weight of the various members in determining the mean behavior and produce also an uncertainty estimate based on the diversity of the results (Potempski et al., 2009; Riccio et al., 2012; Solazzo et al., 2013).

The third phase of the Air Quality Modelling Evaluation International Initiative (AQMEII3) project brought together 14 European and North American modeling groups to simulate the air pollution levels over the two continental areas for the year 2010 (Galmarini et al., 2017). Within AQMEII3, the simulated surface concentrations of health-related air pollutants from each modeling group serve as input to the Economic Valuation of Air Pollution (EVA) model (Brandt et al., 2013a, b). The EVA model is used to calculate the impacts of health-related pollutants on human health over the two continents as well as the associated external costs. EVA model has also been tested and validated for the first time outside Europe. We adopt a MM ensemble approach, in which the outputs of the modeling systems are statistically combined assuming equal contribution from each model and used as input for the EVA model. In addition, the human health impacts (and the associated costs) of reducing anthropogenic emissions, globally and regionally, have been calculated, allowing to quantify the trans-boundary benefits of emission reduction strategies. Finally, following the conclusions of Solazzo and Galmarini (2015), the health impacts have been calculated using an optimal ensemble of models, determined by error minimization. This approach can assess the health impacts with reduced model bias, which we can then compare with the classically derived estimates based on model averaging.

## 2 Material and methods

### 2.1 AQMEII3

#### 2.1.1 Participating models

In the framework of the AQMEII3 project, 14 groups participated in simulating the air pollution levels in Europe and North America for the year 2010. In the present study, we use results from the 13 groups that provided all health-related species (Table 1). As seen in Table 1, six groups have operated the CMAQ model. The main differences among the CMAQ runs reside in the number of vertical levels and horizontal spacing (Table 1), and in the estimation of biogenic emissions. UK1, DE1 and US3 calculated biogenic emissions using the BEIS (Biogenic Emission Inventory System version 3) model, while TR1, UK1 and UK2 calculated biogenic emissions through the Model of Emissions of Gases and Aerosols from Nature (MEGAN) (Guenther et al., 2012). Moreover, DE1 does not include the dust module, while the other CMAQ instances use the inline calculation (Appel et al., 2013), and TR1 uses the dust calculation previously calculated for AQMEII phase 2. Finally, all runs were carried out using CMAQ version 5.0.2, except for TR1, which is based on the 4.7.1 version. The gas-phase mechanisms and the aerosol models used by each group are also presented in Table 1. More details of the model system are provided in the Supplement. The differences in the meteorological drivers and aerosol modules can lead to substantial differences in modeled concentrations (Im et al., 2015b).

#### 2.1.2 Emission and boundary conditions

The base case emission inventories that are used in AQMEII for Europe and North America are extensively described in Pouliot et al. (2015). For Europe, the 2009 inventory of the Netherlands Organisation for Applied Scientific Research Monitoring Atmospheric Composition and Climate (TNO-MACC) anthropogenic emissions was used. In regions not covered by the emission inventory, such as north Africa, five modeling systems have complemented the standard inventory with the HTAPv2.2 datasets (Janssens-Maenhout et al., 2015). For the North American domain, the 2008 National Emission Inventory was used as the basis for the 2010 emissions, providing the inputs and datasets for processing with the SMOKE emissions processing system (Mason et al., 2007). For both continents, the regional-scale emission inventories were embedded in the global-scale inventory (Janssens-Maenhout et al., 2015) used by the global-scale HTAP2 modeling community so as to guarantee coherence and harmonization of the information used by the regional-scale modeling community. The annual totals for European and North American emissions in the HTAP inventory are the same as the MACC and SMOKE emissions. However, there are differences in the temporal distribution, chemical speciation and the vertical distribution used in the models. The C-IFS model (Flemming et al., 2015, 2017) provided chemical boundary conditions. The C-IFS model has been extensively evaluated in Flemming et al. (2015, 2017) and in particular for North America (Hogrefe et al., 2018; Huang et al., 2017). Galmarini et al. (2017) provides more details on the setup of the AQMEII3 and HTAP2 projects.

#### 2.1.3 Model evaluation

The models’ performance in simulating the surface concentrations of the health-related pollutants were evaluated using Pearson’s correlation (*r*), normalized mean bias (NMB), normalized mean gross error (NMGE) and root mean square error (RMSE) to compare the modeled and observed hourly pollutant concentrations over surface measurement stations in the simulation domains. The hourly modeled vs. observed pairs are averaged and compared on a monthly basis. The modeled hourly concentrations were first filtered based on observation availability before the averaging was performed. The observational data used in this study are the same as those in the dataset used in the second phase of AQMEII (Im et al., 2015a, b). Surface observations are provided in the ENSEMBLE system (http://ensemble.jrc.ec.europa.eu/) that is hosted at the Joint Research Centre (JRC). Observational data were originally derived from the surface air quality monitoring networks operating in EU and NA. In EU, surface data were provided by the European Monitoring and Evaluation Programme (EMEP; http://www.emep.int/) and the European Air Quality Database (AirBase; http://acm.eionet.europa.eu/databases/airbase/). In NA, observational data were obtained from the NAtChem (Canadian National Atmospheric Chemistry) database and from the Analysis Facility operated by Environment Canada (http://www.ec.gc.ca/natchem/).

The model evaluation has been conducted for 491 European and 626 North American stations for O_3_, 541 European stations and 37 North American stations for CO, 500 European station and 277 North American stations for SO_2_, and 568 European stations and 156 North American stations for PM_2.5_.

#### 2.1.4 Emission perturbations

In addition to the base case simulations in AQMEII3, a number of emission perturbation scenarios have been simulated (Table 1). The perturbation scenarios feature a reduction of 20 % in the global anthropogenic emissions (GLO) as well as the HTAP2-defined regions of Europe (EUR), North America (NAM) and east Asia (EAS), as explained in detail in Galmarini et al. (2017) and Im et al. (2018). To prepare these scenarios, both the regional models and the global C-IFS model that provides the boundary conditions to the participating regional models have been operated with the reduced emissions. The global perturbation scenario (GLO) reduces the global anthropogenic emissions by 20 %, introducing a change in the boundary conditions as well as a 20 % decrease in the anthropogenic emissions used by the regional models. The North American perturbation scenario (NAM) reduces the anthropogenic emissions in North America by 20 %, introducing a change in the boundary conditions while anthropogenic emissions remain unchanged for Europe, showing the impact of long-range transport for North America, while the scenarios introduce a 20 % reduction of anthropogenic emissions in the HTAP-defined North American region. The European perturbation scenario (EUR) reduces the anthropogenic emissions in the HTAP-defined European domain by 20 %, introducing a change in the anthropogenic emissions while boundary conditions remain unchanged in the regional models, showing the contribution from the domestic anthropogenic emissions only. Finally, the east Asian perturbation scenario (EAS) reduces the anthropogenic emissions in east Asia by 20 %, introducing a change in the boundary conditions while anthropogenic emissions remain unchanged in the regional models, showing the impact of long-range transport from east Asia on the NA concentrations.

### 2.2 Health impact assessment

All modeling groups interpolate their model outputs on a common 0.25° × 0.25° resolution AQMEII grid predefined for Europe (30° W–60° E, 25–70° N) and North America (130–59.5° W, 23.5–58.5° N). All the analyses performed in the present study use the pollutant concentrations on these final grids. Health impacts are first calculated for each individual model, and then the ensemble mean, median and standard deviation are calculated for each health impact. In order to be able to estimate an uncertainty in the health impact calculations, none of the models were removed from the ensemble.

Along with the individual health impact estimates from each model, a multi-model mean dataset (MM_m_, in which all the modeling systems are averaged assuming equally weighted contributions) has been created for each grid cell and time step, hence creating a new model set of results that have the same spatial and temporal resolution of the ensemble-contributing members. In addition to this simple MM_m_, an optimal MM ensemble (MM_opt_) has been generated. MM_opt_ is created following the criteria extensively discussed and tested in the previous phases of the AQMEII activity (Riccio et al., 2012; Kioutsioukis et al., 2016; Solazzo and Galmarini, 2016), where it was shown that there are several ways to combine the ensemble members to obtain a superior model, mostly depending on the feature we wish to promote (or penalize). For instance, generating an optimal ensemble that maximizes the accuracy would require a minimization of the mean error or of the bias, while maximizing the associativity (variability) would require maximizing the correlation coefficient (standard deviation). In this study, the subset of models whose means minimize the mean squared error (MSE) is selected as optimal (MM_opt_). MM_m_ and MM_opt_ have therefore the same spatial resolution with the individual models. The MSE is chosen for continuity with previous AQMEII-related works. The MSE is chosen in light of its property of being composed by bias, variance and covariance types of error, thus lumping together measures of accuracy (bias), variability (variance) and associativity (covariance) (Solazzo and Galmarini, 2016). The minimum MSE has been calculated at the monitoring stations, where observational data are available, and then extended to the entire continental areas. This approximation might affect remote regions away from the measurements. However, considering that for the main pollutants (O_3_ and PM_2.5_) the network of measurements is quite dense around densely populated areas (where the inputs of the MM ensemble are used for assessing the impact of air pollutants on the health of the population), errors due to inaccurate model selection in remote regions might be regarded as negligible (Solazzo and Galmarini, 2015). It should be noted that the selection of the optimal combinations of models is affected by the model’s bias that might stem from processes that are common to all members of the ensemble (e.g., emissions). Therefore, such a common bias does not cancel out when combining the models, possibly creating a biased ensemble. Current work is being devoted to identify the optimal combinations of models from which the offsetting bias is removed (Solazzo et al., 2018).

#### 2.2.1 EVA system

The EVA system (Brandt et al., 2013a, b) is based on the impact-pathway chain (e.g., Friedrich and Bickel, 2001), consisting of the emissions, transport and chemical transformation of air pollutants, population exposure, health impacts and the associated external costs. The EVA system requires hourly gridded concentration input from a regional-scale CTM as well as gridded population data, ERFs for health impacts and economic valuations of the impacts from air pollution. A detailed description of the integrated EVA model system along with the ERFs and the economic valuations used are given in Brandt et al. (2013a).

**Figure 1:**
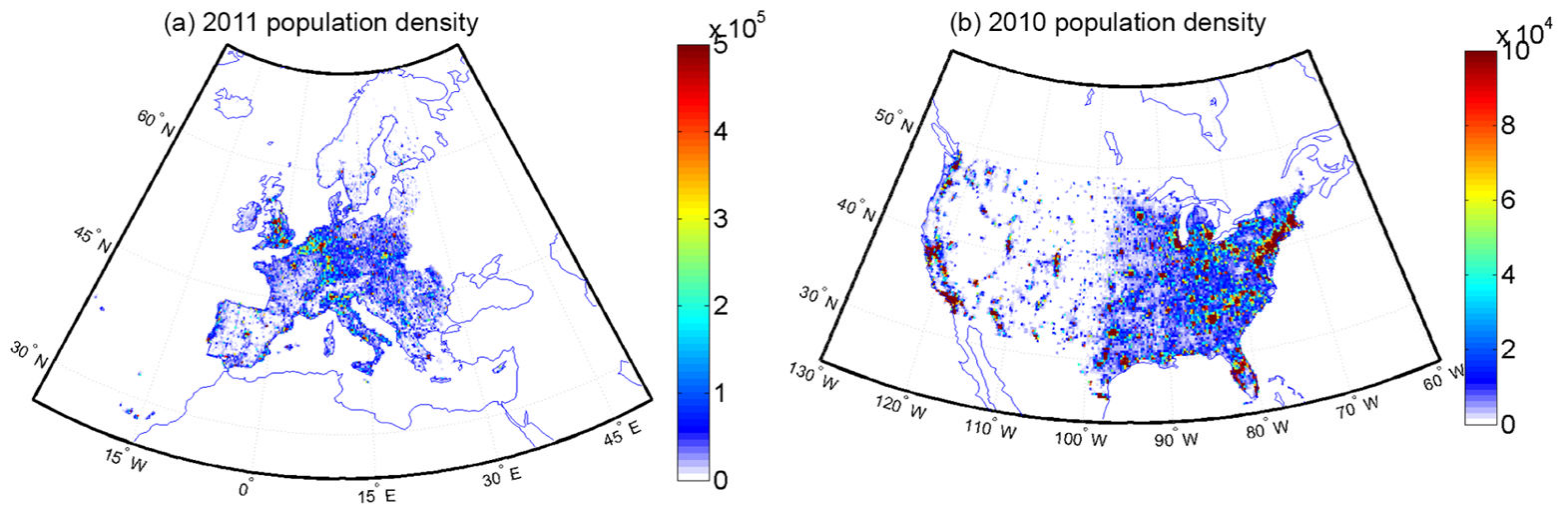
Population density (population per 0.25° × 0.25° grid box) over **(a)** the United States and **(b)** Europe.

The gridded population density data over Europe and the US used in this study are presented in Fig. 1. The population data over Europe are provided on a 1 km spatial resolution from Eurostat for the year 2011 (http://www.efgs.info). The US population data have been provided by the US Census Bureau for the year 2010. The total populations used in this study are roughly 532 and 307 million in Europe and the US, respectively. As the health outcomes are age dependent, the total population data have been broken down to a set of age intervals as follows: babies (under 9 months); children (under 15); and adults above 15, above 30 and above 65. The fractions of population in these intervals for Europe are derived from the Eurostat 2000 database, where the number of persons of each age at each grid cell was aggregated into the above clusters (Brandt et al., 2011), while for the US they are derived from the US Census Bureau for the year 2010 at 5-year intervals.

The EVA system can be used to assess the number of various health outcomes including different morbidity outcomes as well as short-term (acute) and long-term (chronic) mortality, related to exposure of O_3_, CO and SO_2_ (short term) and PM_2.5_ (long term). Furthermore, impact on infant mortality in response to exposure of PM_2.5_ is calculated. The health impacts are calculated using an ERF of the following form:

$$R=\alpha \times \delta_{c}\times P,$$

where *R* is the response (in cases, days or episodes), *c* denotes the pollutant concentration, *P* denotes the affected share of the population, and *α* is an empirically determined constant for the particular health outcome. EVA uses ERFs that are modeled as a linear function, which is a reasonable approximation as showed in several studies (e.g., Pope, 2000; the joint World Health Organization/UNECE Task Force on Health; EU, 2004; Watkiss et al., 2005). Many epidemiological studies have analyzed the concentration–response relationship between ambient PM and mortality using various statistical models. In general, the shapes of the estimated curves did not differ significantly from linear. However, some studies showed non-linear relationships, being steeper at lower than at higher concentrations (e.g., Samoli et al., 2005). Therefore, linear relationships may lead to overestimated health impacts over highly polluted concentration metrics used in each ERF shown in Table 2. The sensitivity of EVA to the different pollutant concentrations is further evaluated in the the Supplement and depicted in Fig. S1. EVA calculates and uses the annual mean concentrations of CO, SO_2_ and PM_2.5_, while for O_3_, it uses the SOMO35 metric that is defined as the yearly sum of the daily maximum of 8 h running average over 35 ppb, following WHO (2013) and EEA (2017).

The morbidity outcomes include chronic bronchitis, restricted activity days, congestive heart failure, lung cancer, respiratory and cerebrovascular hospital admissions, asthma in children (< 15 years) and adults (> 15 years), which includes bronchodilator use, cough and lower respiratory symptoms. The exposure–response functions are broadly in line with estimates derived with detailed analysis in EU-funded research (Rabl et al., 2014; EEA, 2013). To figure out the total number of premature deaths from the years of life lost due to PM_2.5_, they have been converted into lost lives according to a “lifetable” method (explained in detail in Andersen, 2017) but using the factor of 10.6, as reported by Watkiss et al. (2005). To these deaths are added the acute deaths due to O_3_ and SO_2_. The ERFs used, along with their references, in both continents as well as the economic valuations for each health outcome in Europe and the US, respectively, are presented in Table 2. Baseline incidence rates are not assumed to be dissimilar, which is a coarse approach for morbidity. The baseline rates are from Statistics Denmark (http://www.statistikbanken.dk/statbank5a/default.asp?w=1280, last access: 25 April 2018) and lifetables are based on Denmark, which is close to the US and Eurozone average (Andersen, 2017). For a description of the morbidity ERFs, see Andersen et al. (2004, 2008). The economic valuations are provided by Brandt et al. (2013a); see also EEA (2013).

ERFs for all-cause chronic mortality due to PM_2.5_ were based on the findings of Pope et al. (2002), which is the most extensive study available, following conclusions from the scientific review of the Clean Air For Europe (CAFE) program (Hurley et al., 2005; Krupnick et al., 2005). The results from Pope et al. (2002) are further supported by Krewski et al. (2009) and more recently by the latest HRAPIE project report (WHO, 2013a). Therefore, as recommended by WHO (2013a), EVA uses the ERFs based on the meta-analysis of 13 cohort studies as described in Hoek et al. (2013). In EVA, the number of lost life years for a Danish population cohort with normal age distribution, when applying the ERF of Pope et al. (2002) for all-cause mortality (relative risk, RR of 1.062 (1.040–1.083) on a 95 % confidence interval), and the latency period indicated, sums to 1138 years of life lost (YOLL) per 100 000 individuals for an annual PM_2.5_ increase of 10 μg m^−3^ (Andersen et al., 2008). EVA uses a counterfactual PM_2.5_ concentration of 0 μg m^−3^ following the EEA methodology, meaning that the impacts have been estimated for the full range of modeled concentrations from 0 μg m^−3^ upwards. Applying a low counterfactual concentration can underestimate health impacts at low concentrations if the relationship is linear or close to linear (Anenberg et al., 2015). However, it is important to note that uncertainty in the health impact results may increase at low concentrations due to sparse epidemiological data. Assuming linearity at very low concentrations may distort the true health impacts of air pollution in relatively clean atmospheres (Anenberg et al., 2016).

It has been shown that O_3_ concentrations above the level of 35 ppb involve an acute mortality increase, presumably for weaker and elderly individuals. EVA applies the ERFs selected in CAFE for post-natal deaths (age group 1–12 months) and acute deaths related to O_3_ (Hurley et al., 2005). WHO (2013a) also recommends the use of the daily maximum of 8 h mean O_3_ concentrations for the calculation of the acute mortality due to O_3_. There are also studies showing that SO_2_ is associated with acute mortality, and EVA adopts the ERF identified in the APHENA study – Air Pollution and Health: A European Approach (Katsouyanni et al., 1997).

Chronic exposure to PM_2.5_ is also associated with morbidity, such as lung cancer. EVA employs the specific ERF (RR of 1.08 per 10 μg m^−3^ PM_2.5_ increase) for lung cancer indicated in Pope et al. (2002). Bronchitis has been shown to increase with chronic exposure to PM_2.5_ and we apply an ERF (RR of 1.007) for new cases of bronchitis based on the AHSMOG study (involving non-smoking Seventh-Day Adventists; Abbey et al., 1999), which is the same epidemiological study as in CAFE (Abbey et al., 1995; Hurley et al., 2005). The ExternE crude incidence rate was chosen as a background rate (ExternE, 1999), which is in agreement with a Norwegian study, rather than the pan-European estimates used in CAFE (Eagan et al., 2002). Restricted activity days (RADs) comprise two types of responses to exposure: so-called minor restricted activity days as well as work-loss days (Ostro, 1987). This distinction enables accounting for the different costs associated with days of reduced well-being and actual sick days. It is assumed that 40 % of RADs are work-loss days based on Ostro (1987). The background rate and incidence are derived from ExternE (1999). Hospital admissions are deducted to avoid any double counting. Hospital admissions and health effects for asthmatics (here corresponding to the responses of bronchodilator use, cough and lower respiratory symptoms) are also based on ExternE (1999).

Table 2 lists the specific valuation estimates applied in the modeling of the economic valuation of mortality and morbidity effects. A principal value of EUR 1.5 million was applied for preventing an acute death, following expert panel advice (EC, 2001). For the valuation of a life year, the results from a survey relating specifically to air pollution risk reductions were applied (Alberini et al., 2006), implying a value of EUR 57 500 per year of life lost (YOLL). With the more conservative metric of estimating lost life years, rather than “full” statistical lives, there is no adjustment for age. This is due to the fact that government agencies in Europe, including the European Commission, apply a methodology for costs of air pollution that is based on accounting for lost life years, rather than for entire statistical lives as is customary in USA. While the average traffic victim, for instance, is middle-aged and likely to lose about 35–40 years of life expectancy, pollution victims are believed to suffer significantly smaller losses of years (EAHEAP, 1999; Friedrich and Bickel, 2001). To avoid overstating the benefits of air pollution control, these are treated as proportional to the number of life years lost. Most of the excess mortality is due to chronic exposure to air pollution over many years, and the life year metric is based on the number of lost life years in a statistical cohort. Following the guidelines of the Organisation for Economic Co-operation and Development (OECD, 2006), the predicted acute deaths, mainly from O_3_, are valuated here with the adjusted value for preventing a fatality (VSL, value of a statistical life). The lifetables are obtained from European data and are applied to the US as the average life expectancy in the US is similar to that in Europe and close to the OECD average (OECD, 2016). The willingness to pay for reductions in risk obviously differs across income levels. However, in the case of air pollution costs, adjustment according to per capita income differences among different states is not regarded as appropriate, because long-range transport implies that emissions from one state will affect numerous other states and their citizens. The valuations are thus adjusted with regional purchasing power parities (PPPs) of EU27 and USA.

Cost–benefit analysis in the US related to air pollution proceeds from a standard approach, where abatement measures preventing premature mortality are considered according to the number of statistical fatalities avoided, which are appreciated according to the VSL (presently USD 7.4 million). In contrast, and following recommendations from the UK working group on Economic Appraisal of the Health Effects of Air Pollution (EAHEAP, 1999), focus in EU has been on the possible changes in average life expectancy resulting from air pollution. In EU, the specific number of life years lost as a result of changes in air pollution exposures is estimated based on lifetable methodology and monetized with value-of-life-year (VOLY) unit estimates (Holland et al., 1999; Leksell and Rabl, 2001). The theoretical basis is a lifetime consumption model according to which the preferences for risk reduction will reflect expected utility of consumption for remaining life years (Hammitt, 2007; OECD, 2006, p. 204). The much lower VSL values customary in Europe (presently EUR 2.2 million) add decisively to the differences, as VOLY is deducted from this value. By using a common valuation framework according to the EU approach, we allow for direct comparisons of the monetary results. It follows from OECD recommendations (2012) to correct with PPP when doing such benefit transfer. The unit values have been indexed to 2013 prices as indicated in Table 2.

## 3 Results

### 3.1 Model evaluation

**Figure 2:**
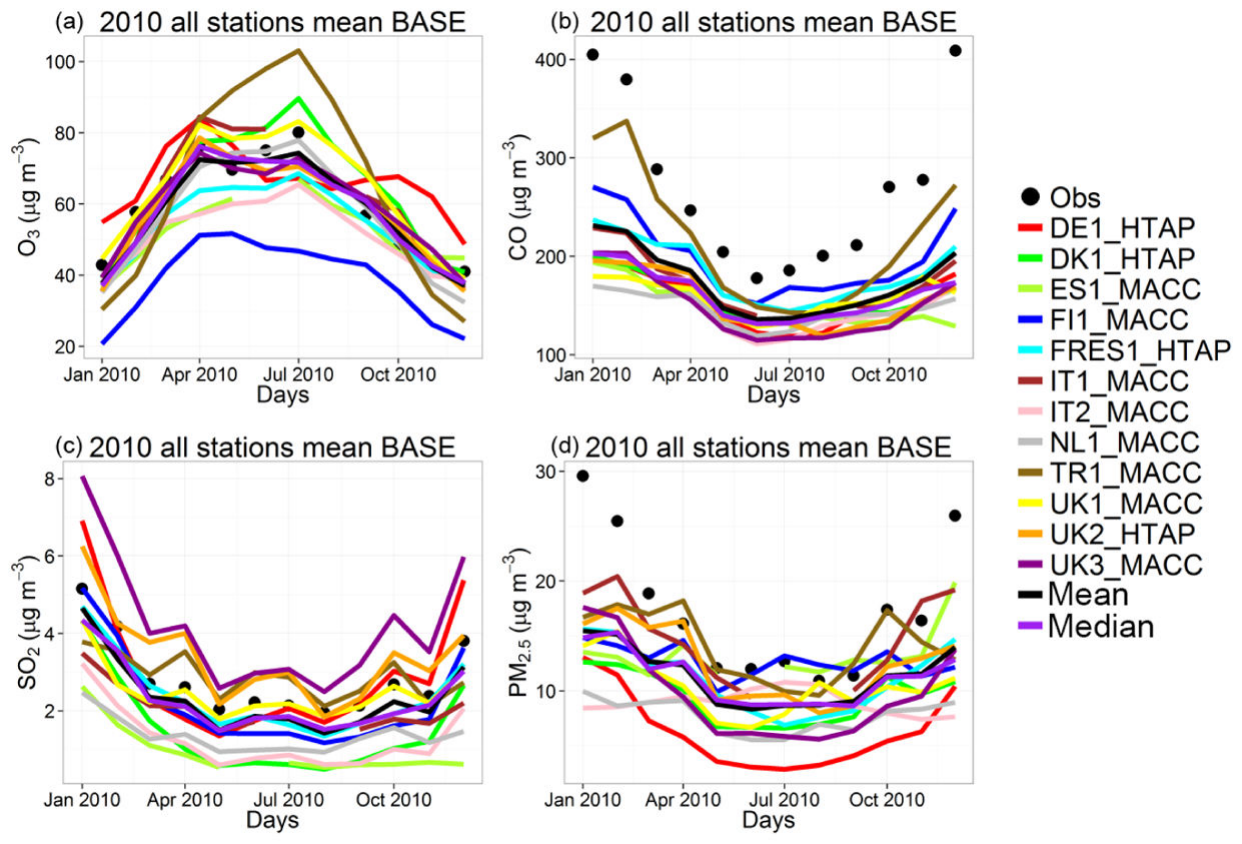
Observed and simulated (base case) monthly **(a)** O_3_, **(b)** CO, **(c)** SO_2_ and **(d)** PM_2.5_ concentrations over Europe.

**Figure 3:**
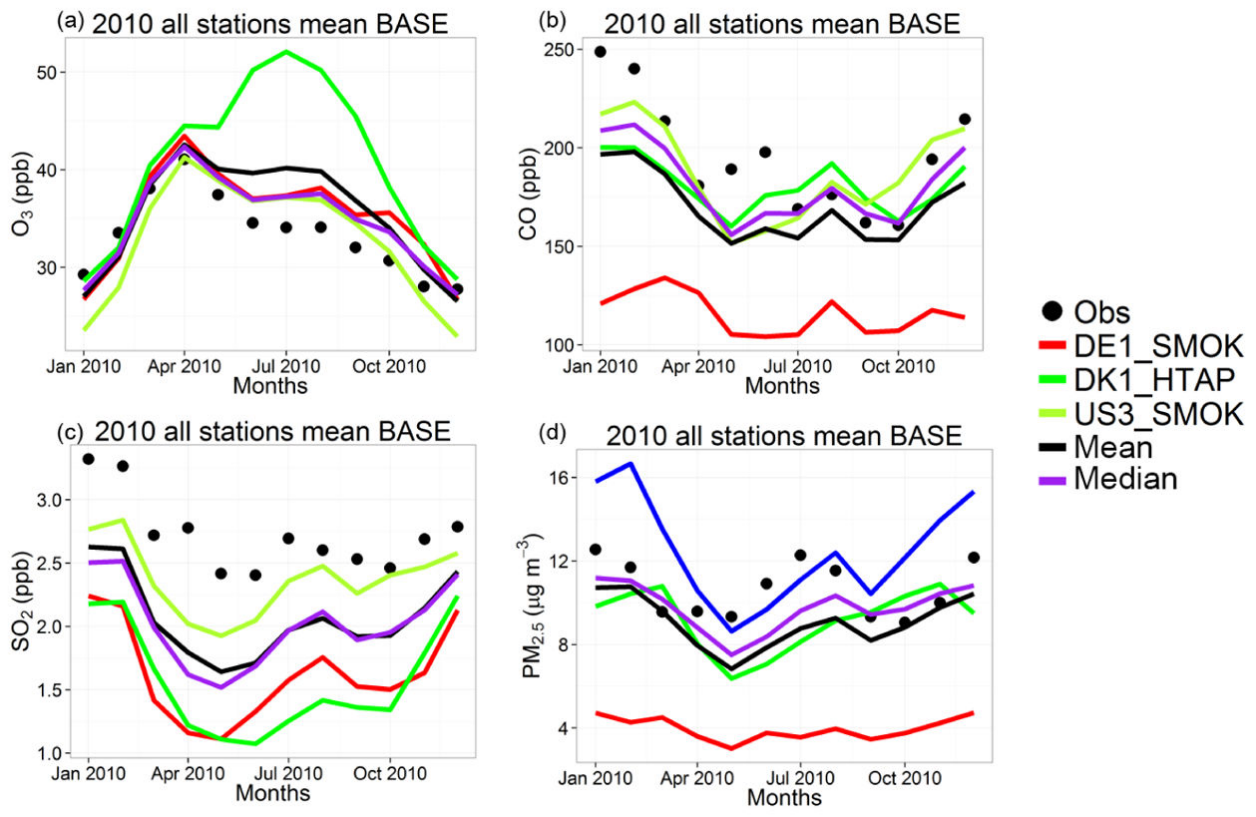
Observed and simulated (base case) monthly **(a)** O_3_, **(b)** CO, **(c)** SO_2_ and **(d)** PM_2.5_ concentrations over the US.

Observed and simulated hourly surface O_3_, CO, SO_2_ and daily PM_2.5_, which are species used in the EVA model to calculate the health impacts, over Europe and North America for the entire 2010 were compared in order to evaluate each model’s performance. The statistical parameters to evaluate the models and their equations are provided in the Supplement. For a more thorough evaluation of models and species, see Solazzo et al. (2017). The results of this comparison are presented in Table S1 for EU and NA, along with the multi-model mean and median values. The monthly time series plots of observed and simulated health-related pollutants are also presented in Figs. 2 and 3. The monthly means are calculated using the hourly pairs of observed and modeled concentrations at each station. The results show that, over Europe, the temporal variability of all gaseous pollutants is well captured by all models with correlation coefficients (*r*) higher than 0.70 in general. The NMBs in simulated O_3_ levels are generally below 10 % with few exceptions up to −35 %. CO levels are underestimated by up to 45 %, while the majority of the models underestimated SO_2_ levels by up to 68 %, while some models overestimated SO_2_ by up to 49 %. PM_2.5_ levels are underestimated by 19 to 63 %. Over Europe, the median of the ensemble performs better than the mean in terms of model bias (NMB) for O_3_ (by 52 %), while for CO, SO_2_ and PM_2.5_, the mean performs slightly better than the median (Table S1).

**Figure 4:**
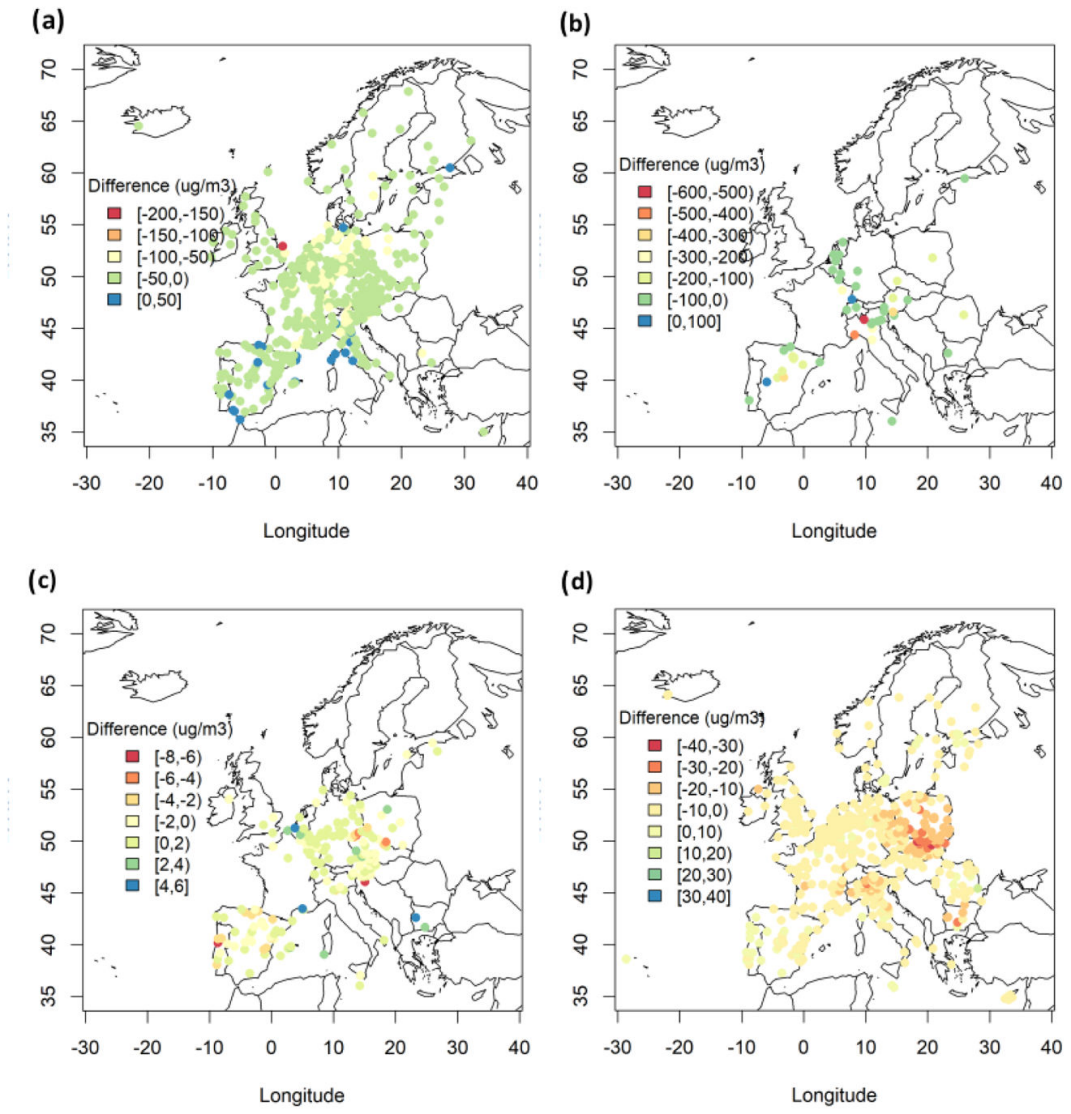
Spatial distribution of annual MM mean bias (μg m^−3^) for **(a)** DM8H O_3_, **(b)** CO, **(c)** SO_2_ and **(d)** PM_2.5_ over Europe.

We have further evaluated the models’ performance in simulating the annual mean pollutant levels over individual measurements stations and plotted the geographical distribution of the bias. Figure 4 presents the multi-model mean geographical distribution of bias from daily max 8 h (DM8H) average O_3_, CO, SO_2_ and PM_2.5_ over Europe, while Figs. S2–S5 show annual mean bias for O_3_, CO, SO_2_ and PM_2.5_ for each model, respectively. DM8H O_3_ levels over Europe are generally underestimated by up to 50 μg m^−3^, with few overestimations up to 50 μg m^−3^ over southern Europe (Fig. 4a). The geographical pattern of annual mean O_3_ bias is similar among the models with slight differences (±10 μg m^−3^) in the bias (Fig. S2). CO levels are underestimated over all stations by up to 600 μg m^−3^ except for few stations where CO levels are overestimated by up to 100 μg m^−3^ (Fig. 4b). All models underestimated CO levels over the majority of the stations (Fig. S3). SO_2_ levels are slightly overestimated over central and southern Europe (Fig. 4c). There are also underestimations over few stations with no specific geographical pattern. Similar to CO, all models underestimated SO_2_ levels over the majority of the stations (Fig. S4). Finally, PM_2.5_ levels are underestimated by up to 10 μg m^−3^ over most of Europe (Fig. 4d), with larger underestimations over eastern Europe up to 30 μg m^−3^.

Over North America, the hourly O_3_ variation is well captured by all models (Table S1), with DK1 having slightly lower *r* coefficient compared to the other models and largest NMB (Fig. 3a). The hourly variations of CO and SO_2_ levels are simulated with relatively lower *r* values (Fig. 3b, c), with SO_2_ levels having the highest underestimations. The PM_2.5_ levels are underestimated by ~ 15 % except for the DE1 model, having a large underestimation of 63 % (Table S1). As DE1 and US3 use the same SMOKE emissions and CTM, the large difference in PM_2.5_ concentrations can be partly due to the differences in horizontal and vertical resolutions in the model setups, as can also be seen in the differences in the CO concentrations. There are also differences in the aerosol modules and components that each model simulates. For example, DE1 uses an older version of the secondary organic aerosol (SOA) module, producing ~ 3 μg m^−3^ less SOA, which can explain ~ 20 % of the bias over North America. Over the North American domain, the median outscores the mean for O_3_ (by 35 %), CO (by 52 %) and PM_2.5_ (by 29 %), while for SO_2_, the median produces 26 % higher NMB compared to the mean. The DK1 model simulates a much higher bias for O_3_ and SO_2_ compared to other models in the North American domain, while DE1 has the largest bias for CO and PM_2.5_.

**Figure 5:**
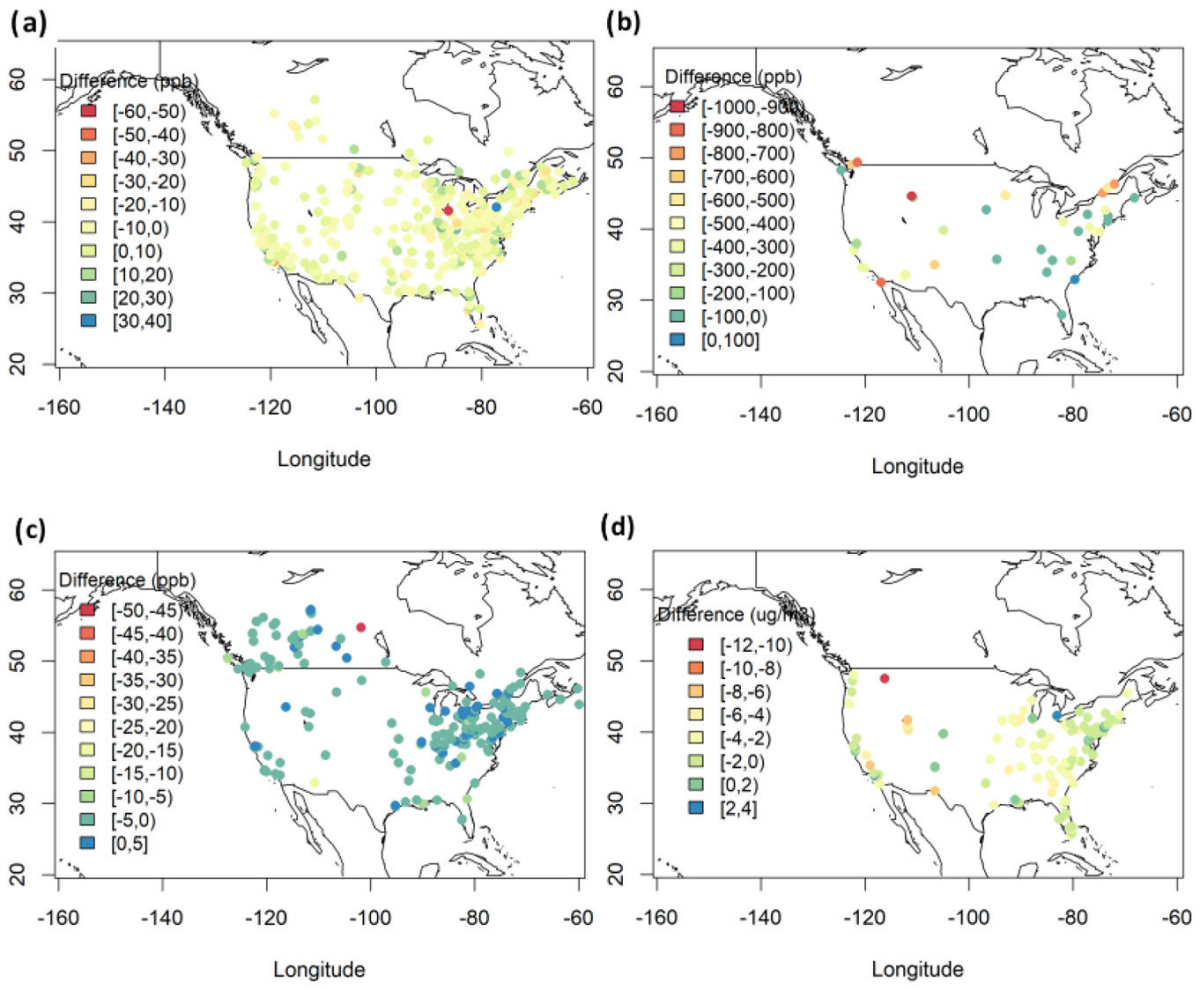
Spatial distribution of annual MM mean bias (ppb for gases and μg m^−3^ for PM_2.5_) for **(a)** DM8H O_3_, **(b)** CO, **(c)** SO_2_ and **(d)** PM_2.5_ over North America.

DM8H O_3_ levels are generally underestimated by the MM mean over the US by up to 20 ppb, while over the eastern and central US there are also overestimations by up to 10 ppb (Fig. 5a). As seen in Fig. S6, all three models have very similar performance over the US, with DK1 simulating a slightly lower underestimation and a higher overestimation compared to DE1 and US3. DE1 and DK1 have very similar spatial pattern in terms of CO bias, in particular over the eastern coast of the US (Fig. S7). CO levels are underestimated by ~ 100 ppb over the majority of the stations, especially over the eastern US, while there are much larger underestimations over the western US by up to 1000 ppb (Fig. 5b). SO_2_ levels are underestimated by up to 5 ppb over the majority of the stations in the US, with few overestimations of up to 5 ppb (Fig. 5c). DE1 and DK1 have a very similar spatial distribution of bias, while US3 has slightly more overestimations (Fig. S8). Finally, PM2.5 levels are underestimated over majority of the stations by up to 6 μg m^−3^, with few overestimations by 2–4 μg m^−3^ (Fig. 5d). DE1 has the largest underestimations compared to DK1 and US3 (Fig. S9).

Table S1 shows that the ensemble median performs slightly better than the ensemble mean for all pollutants over both continents in terms of the bias and error, while the difference in *r* is rather small. Over the European stations, the median has improved results over the mean by up to 14 % for *r* and up to 9 % for the RMSE. The improvements in *r* over the US are much smaller compared to Europe (up to ~ 4 %), while the RMSE is improved by up to 27 %, except for SO_2_ where the median has 14 % higher RMSE than the mean.

### 3.2 Health outcomes and their economic valuation in Europe

The different health outcomes calculated by each model in Europe as well as their multi-model mean and median are presented in Table S2. Table 3 presents the mean of the individual model estimates as MM_mi_. Standard deviations calculated from the individual model estimates are presented along with the MM_mi_ in the text. The health impact estimates vary significantly between different models. The different estimates obtained are found to vary up to a factor of 3. Among the different health outcomes, the individual models simulated the number of congestive heart failure (CHF) cases to be between 19 000 and 41 000 (mean of all individual models, MM_mi_, 31 000 ± 6500). The number of lung cancer cases due to air pollution is calculated to be between 30 000 and 78 000 (mean of all individual models, MM_mi_, 55 000 ± 14 000). Finally, the total (acute and chronic) number of premature deaths due to air pollution is calculated to be 230 000 to 570 000 (mean of all individual models, MM_mi_, 414 000 ± 100 000). The health impacts calculated as the median of individual models differ slightly (~ ±1 %) from those calculated as the mean of individual models (Table S2) due to the slight differences in the model bias (NMB) and error (NMGE and RMSE) between the mean and the median performance statistics of the models.

In addition to averaging the health estimates from individual models (MM_mi_), we have also produced a multi-model mean concentration data (MM_m_) by taking the average of concentrations of each species calculated by all models at each grid cell and hour, and feeding it to the EVA model. We have calculated the number of premature death cases in Europe (Table 3) using MM_m_. The difference in the health impacts calculated using MM_m_ data from the mean of all individual model (MM_mi_) estimates is smaller than 1 %. The number of premature death cases in Europe as calculated as the average of all models in the multi-model ensemble, MM_mi_, due to exposure to O_3_ is 12 000 ± 6500, while the cases due to exposure to PM_2.5_ are calculated to be 390 000 ± 100 000 (180 000–550 000). The O_3_-related mortality well agrees with Liang et al. (2018), who used the multi-model mean of the HTAP2 global model ensemble, which calculated an O_3_-related mortality of 12 800 (600–28 100). The multi-model mean (MM_mi_) PM_2.5_-related mortality in the present study is much higher than that in the HTAP2 study: 195 500 (4400–454 800). The results also agree with the most recent EEA findings (EEA, 2015), which calculated a total of 419 000 premature deaths due to O_3_ and PM_2.5_ in the EU28 countries. There is also agreement with Geels et al. (2015), who calculated 388 000 premature death cases in Europe for the year 2000. This difference can be attributed to the number of mortality cases as calculated by the individual models, where the HTAP2 ensemble calculates a much lower minimum while the higher ends from the two ensembles agree well.

The differences between the health outcomes calculated by the HTAP2 and AQMEII ensembles arise firstly from the differences in the concentration fields due to the differences in models, in particular spatial resolutions as well as the gas and aerosol treatments in different models, but also the differences in calculating the health impacts from these concentration fields. EVA calculates the acute premature deaths due to O_3_ by using the SOMO35 metric. On the other hand, in HTAP2, O_3_-related premature deaths are calculated by using the 6-month seasonal average of daily 1 h maximum O_3_ concentrations. Both groups use the annual mean PM_2.5_ to calculate the PM_2.5_-related premature deaths. In addition to O_3_ and PM_2.5_, EVA also takes into account the health impacts from CO and SO_2_, which are missing in the HTAP2 calculations.

Among all models, the DE1 model calculated the lowest health impacts for most health outcomes, which can be attributed to the largest underestimation of PM_2.5_ levels (NMB of −63 %; Table S2) due to lower spatial resolution of the model that dilutes the pollution in the urban areas, where most of the population lives. The number of premature deaths calculated by this study is in agreement with previous studies for Europe using the EVA system (Brandt et al., 2013a; Geels et al., 2015). Recently, EEA (2015) estimated that air pollution is responsible for more than 430 000 premature deaths in Europe, which is in good agreement with the present study.

**Figure 6:**
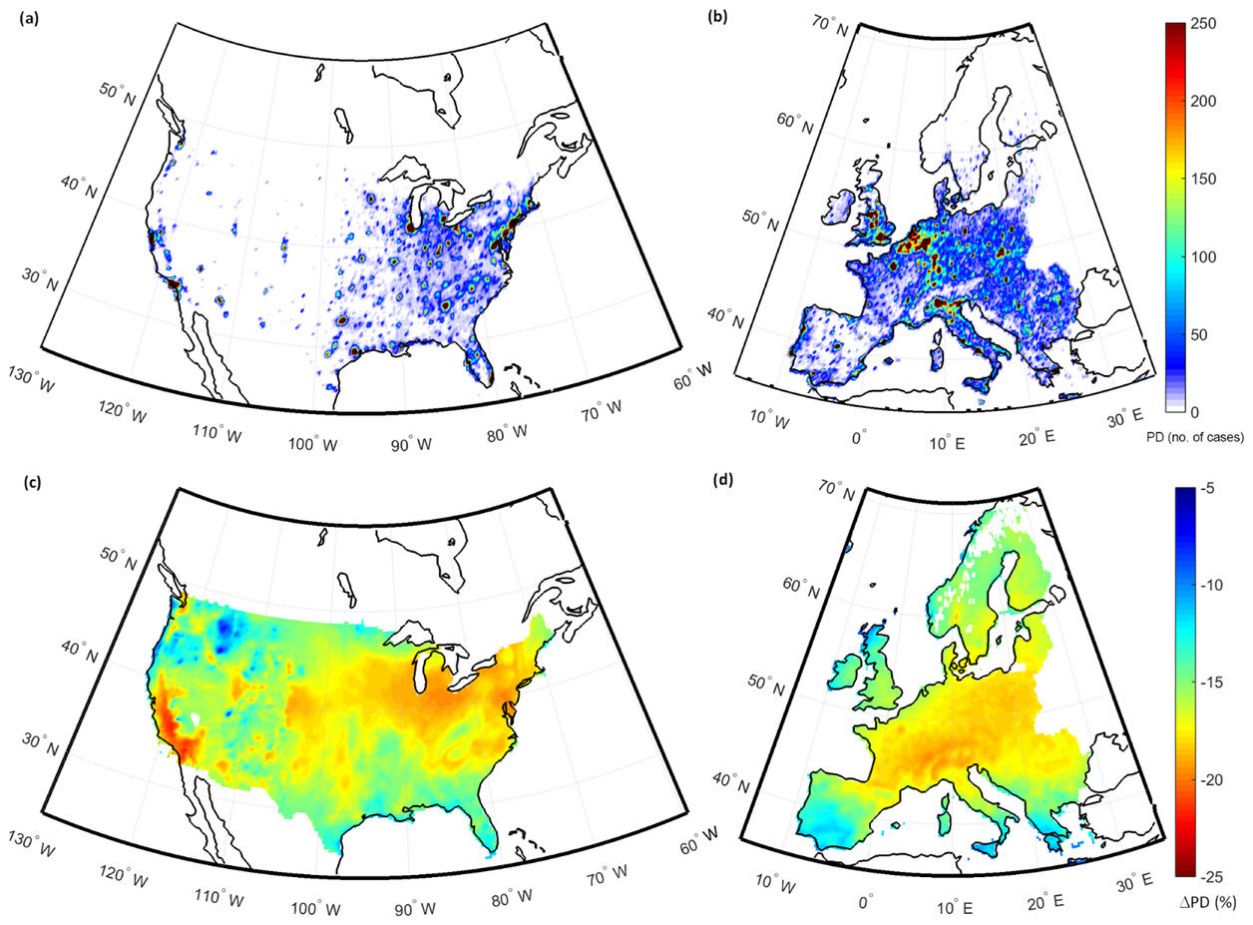
Spatial distribution of the number of total premature deaths (PD: units in number of cases per 0.25° × 0.25° grid box) in **(a)** the United States and **(b)** Europe, and the relative change (%) in the number of premature deaths in response to the GLO scenario in **(c)** the United States and **(d)** Europe in 2010 as calculated by the multi-model mean ensemble.

Figure 6a presents the geographical distribution of the number of premature deaths in Europe in 2010. The figure shows that the number of cases is strongly correlated with the population density (Fig. 1a), with the largest numbers seen in the Benelux and Po Valley regions that are characterized as the pollution hot spots in Europe as well as in megacities such as London, Paris, Berlin and Athens.

The economic valuation of the air-pollution-associated health impacts calculated by the different models, along with their mean and median, is presented in Table 4. A total cost of EUR 196 billion to 451 billion (MM mean cost of EUR 300 ± 70 billion) was estimated over Europe (EU28). Results show that 5 % (1–11 %) of the total costs are due to exposure to O_3_, while 89 % (80–96 %) are due to exposure to PM_2.5_. Brandt et al. (2013a) calculated a total external cost of EUR 678 billion for the year 2011 for Europe, larger than the estimates of this study, which can be explained by the differences in the simulation year and the emissions used in the models as well as the countries included in the two studies (the previous study includes, e.g., Russia).

### 3.3 Health outcomes and their economic valuation in the US

The different health outcomes calculated by each model for the US as well as their mean and median are presented in Table S2. The variability among the models (~ 3) is similar to that in Europe. The number of congestive heart failure cases in the US as calculated as the average of all models in the ensemble (MM_mi_) is calculated to be 13 000 (7000–18 000), while the lung cancer cases due to air pollution are calculated to be 22 000 (9000–31 000). Finally, the number of premature deaths due to air pollution is calculated to be 165 000 ± 75 000, where 25 000 ± 6000 cases are calculated due to exposure to O_3_ and 140 000 ± 72 000 cases due to exposure to PM_2.5_. The MM_m_ dataset leads to a total of 149 000 premature deaths that is 6 % smaller than the average estimate from individual models (MM_mi_). Due to the large reduction of NMB by the median compared to the mean of individual models (Table S1), the multi-model health impacts calculated as the median of health impacts from individual models are ~ 13 % higher than the health impacts calculated from the MM_mi_. The O_3_ and PM_2.5_ mortality cases as calculated by the AQMEII and HTAP2 model ensembles reasonably agree. Liang et al. (2018) calculated an O_3_-related mortality of 14 700 (900–30 400) and a PM_2.5_-related mortality of 78 600 (4500–162 600). These results are in very good agreement with the US EPA (2011) estimates of total number of 160 000 premature death cases in the year 2010 and with Caiazzo et al. (2013), who calculated 200 000 premature death cases from combustion sources in the US. Among all models, the DE1 model calculated the lowest health impacts for most health outcomes, which can be attributed to the largest underestimation of PM_2.5_ levels (NMB of −63 %; Table S2).

The premature death cases in North America are mostly concentrated over the New York area, as well as in hot spots over Chicago, Detroit, Houston, Los Angeles and San Francisco (Fig. 6b). The figure shows that the number of cases is following the pattern of the population density. The economic valuation of the air-pollution-associated health impacts calculated by the different models in the US is shown in Table 4. As seen in the table, a total cost of ~ EUR 145 billion is calculated. Results show that ~ 22 % of the total costs are due to exposure to O_3_ while ~ 78 % are due to exposure to PM_2.5_. The major health impacts in terms of their external costs are slightly different in North America compared to Europe.

### 3.4 Health impacts and their economic valuation through optimal reduced ensemble subset

The effect of pollution concentrations (EVA input) on health impacts (EVA output) is investigated in order to estimate the contribution of each air pollutant in the EVA system to health impacts over different concentration levels. The technical details are provided in the Supplement.

Results show that for the particular input (gridded air pollutant concentrations from individual model) to output (each health outcome) configuration, the PM_2.5_ drives the variability of the different health impact and at least 81 % of the variation of the health impacts are explained by sole variations in the pollutants (i.e., without interactions; Table S3). Table S1 also shows that the most important contribution to the health impacts is from PM_2.5_, followed by CO and O_3_ (with much smaller influence though). The impact of perturbing PM_2.5_ by a fixed fraction of its standard deviation on the health impact is roughly double compared to CO and O_3_.

We have run the EVA system over an all-model mean (MM_m_) dataset and an optimal reduced ensemble dataset (MM_opt_) calculated for each of the pollutants in the two domains in order to see how and whether an optimal reduced ensemble changes the assessment of the health impacts compared to an all-model ensemble mean. Table 5 shows some sensible error reduction, although the temporal and spatial averages mask the effective improvement in accuracy from MM_m_ to MM_opt_. In Europe, the optimal reduced ensemble decreases the RMSE by up to 24 %, while in NA, the error reduction is much larger (4 to 147 %). On a seasonal basis, MM_opt_ reduces RMSE in PM_2.5_ over Europe by 23 % in winter, while smaller decreases are achieved in other seasons (~ 10 %). Regarding O_3_, improvement is 16–22 %, with the largest improvement in spring. In NA, the improvement in winter RMSE in PM_2.5_ is smallest (~ 2 %), while larger improvements are achieved in other seasons (~ 7 to ~ 9 %). For O_3_, the largest RMSE reduction in NA is achieved for the summer period by 14 %.

The analysis of the aggregated health indices’ data for Europe (Table S1) shows that EVA indices rely principally on the PM_2.5_ levels and then on the CO and O_3_ values. Therefore, the relative improvement of the indices with the optimal ensemble should be proportional to the relative improvement in PM_2.5_, CO and O_3_. The proportionality rate for each pollutant is given in Table S3, assuming all pollutants are varied (from MM_m_ to MM_opt_) away from their mean by the same fraction of their variance. As seen in the Table 3, from MM_m_ to MM_opt_, the health indices increase by up to 30 % in Europe. This increase is due to a 27 % increase in the domain-mean PM_2.5_ levels when the optimal reduced ensemble is used, as well a slight increase in O_3_ by ~ 1 %. The number of premature deaths in Europe increase from 410 000 to 524 000 (28 %), resulting in a much higher estimate compared to previous mortality studies. On the contrary, in the US, the mean PM_2.5_ and O_3_ levels decrease from 2.94 to 2.62 μg m^−3^ (~ 11 %) and 18.7 to 18.4 ppb (~ 2 %), respectively. In response, the health indices decrease by ~ 11 % (Table 3). The number of premature death cases in NA decreases from 149 000 to 133 000.

### 3.5 Impact of anthropogenic emissions on the health impacts and their economic valuation

The impacts of emission perturbations on the different health outcomes over Europe and the US as calculated by the individual models are presented in Tables S4–S6. Table 6 shows the impacts of the different emission perturbations on the premature death cases in Europe and the US as calculated by a subset of models that simulated the base case and all three perturbation scenarios (MM_c_). Results show that, in Europe, the 20 % reduction in the global anthropogenic emissions leads to ~ 17 % domain-mean reduction in all the health outcomes, with a geographical variability as seen in Fig. 6c. The figure shows that the larger changes in mortality are calculated in the central and northern parts of Europe (15–20 % decreases), while the changes are smaller in the Mediterranean region (5–10 %), highlighting the non-linearity of the response to emission reductions. However, it should be noted that global models or coarse-resolution regional models (as in this study) cannot capture the urban features and pollution levels, and thus non-linearities should be addressed further using fine spatial resolutions or urban models. The models vary slightly, simulating the response to the 20 % reduction in global emissions, estimating decreases of ~ 11 to 20 %. The number of premature deaths decreased on average by ~ 50 000, ranging from −39 000 (DK1) to −103 000 (IT1). This number is in good agreement with the ~ 45 000 premature deaths calculated by the HTAP2 global models (Liang et al., 2018). The MM_c_ ensemble calculated 15 and 17 % decreases in the O_3_- and PM_2.5_-related premature death cases, respectively, in response to the GLO scenario. This decrease in the global anthropogenic emissions leads to an estimated decrease of EUR 56 ± 18 billion in associated costs in Europe (Table 6).

As seen in Table 6, a 20 % reduction of anthropogenic emissions in the EUR region, as defined in HTAP2, avoids 47 000 premature deaths, while a 20 % reduction of the anthropogenic emissions in the NAM region leads to a much smaller decrease of premature deaths in Europe (~ 1000). These improvements in the number of premature deaths are in agreement with a recent HTAP2 global study that calculated reductions of ~ 34 000 and ~ 1000 for the EUR and NAM scenarios, respectively (Liang et al., 2018), and with Anenberg et al. (2009, 2014), which amounts to a sum of avoided premature deaths being ~ 39 000 and 1800 as calculated by the MM mean. Both the global and regional models agree that the largest impacts of reducing emissions with respect to premature deaths come from emissions within the source region, while foreign sources contribute much less to improvements in avoiding adverse impacts of air pollution. The decreases in health impacts in the EUR and NAM scenarios correspond to decreases in the associated costs by EUR −47 ± 16 billion and EUR −1.4 ± 0.4 billion, respectively. This is consistent with results in Brandt et al. (2012), where a contribution of ~ 1 % to PM_2.5_ concentrations in Europe originates from the NAM region.

The 20 % reduction in global anthropogenic emissions leads to 18 % reduction in the health outcomes (Table 6) in the US, with a geographical variability in the response. Figure 6d shows that the largest decreases in mortality are calculated for the western coast of the US (~ 20 %) and there is a slightly lower response in the central and eastern parts of the US (15–20 %). The number of premature death cases, as calculated by the mean of all individual models, decreases from ~ 160 000 ± 70 000 to ~ 130 000 ± 60 000, avoiding EUR 24 ± 10 billion (Table 6) in external costs, also in agreement with the ensemble of HTAP2 global models (~ 23 000) The O_3_-related premature death cases decreased by 42 %, while the PM_2.5_-related cases decreased by 18 %.

A 20 % reduction of the North American emissions avoids ~ 25 000 ± 12 000 premature deaths (−16 %), suggesting that ~ 80 % of avoided premature deaths are achieved by reductions within the source region, while 20 % (~ 5000 premature deaths) are from foreign sources. This number is also in good agreement with Liang et al. (2018), who estimated a reduction of premature deaths of ~ 20 000 due to O_3_ and PM_2.5_ in the United States due to an emission reduction of 20 % within the region itself, using the ensemble mean of the HTAP2 global models. These results are much larger than the number of avoided premature deaths of ~ 11 000 as calculated by the sum of Anenberg et al. (2009, 2104). The corresponding benefit is calculated to be EUR 21 ± 9 billion in the NAM scenario. According to results from the EAS scenario, among these 5000 avoided cases that are attributed to the foreign emission sources, 1900 ± 2000 premature deaths can be avoided by a 20 % reduction of the east Asian emissions, avoiding EUR 2.5 ± 3 billion. Our number of avoided premature deaths due to the EAS scenario is much higher than 580 avoided premature deaths calculated by Liang et al. (2018) and 380 avoided cases as calculated by Anenberg et al. (2009 and 2014).

## 4 Conclusions

The impact of air pollution on human health and its economic valuation for the society across Europe and the United States are modeled by a multi-model ensemble of regional models from the AQMEII3 project. All regional models used boundary conditions from the C-IFS model and emissions from either the MACC inventory in Europe or the EPA inventory for the North America, or the global inventory from HTAP. Sensitivity analysis on the dependence of models on different sets of boundary conditions has not been conducted so far but large deviations from the current results in terms of health impacts are not expected. The modeled surface concentrations by each individual model are used as input to the EVA system to calculate the resulting health impacts and the associated external costs from O_3_, CO, SO_2_ and PM_2.5_. Along with a base case simulation for the year 2010, some groups performed additional simulations, introducing 20 % emission reductions both globally and regionally in Europe, North America and east Asia.

The base case simulation of each model is evaluated with available surface observations in Europe and North America. Results show large variability among models, especially for PM_2.5_, where models underestimate by ~ 20 to ~ 60 %, introducing a large uncertainty in the health impact estimates as PM_2.5_ is the main driver for health impacts. The differences in the models are largely due to differences in the spatial and vertical resolutions, meteorological inputs, inclusion of natural emissions, dust (in particular), as well as missing or underestimated SOA mass, which is critical for the PM_2.5_ mass. As shown in the Supplement, the CTMs diverge a lot on the representation of particles and their size distribution, SOA formation, as well as the inclusion of natural sources. As the anthropogenic emissions are harmonized in the models, they represent a minor uncertainty in terms of model-to-model variation. However, differences in the treatment of the temporal, vertical and chemical distributions of the particulate and volatile organic species have an influence in the model calculations and therefore lead to model-to-model variations.

The variability of health impacts among the models can be up to a factor of 3 in Europe (12 models) and the US (3 models) among the different health impacts. The multi-model mean total number of premature deaths is calculated to be 414 000 in Europe and 160 000 in the US, where PM_2.5_ contributes by more than 90 %. These numbers agree well with previous global and regional studies for premature deaths due to air pollution. In order to reduce the uncertainty coming from each model, an optimal ensemble set is produced, that is, the subset of models that produce the smallest error compared to the surface observations at each time step. The optimum ensemble results in an increase of health impacts by up to 30 % in Europe and a decrease by ~ 11 % in the United States. These differences clearly demonstrate the importance of the use of optimal reduced multi-model ensembles over traditional all-model mean ensembles, both in terms of scientific results but also in policy applications.

Finally, the role of domestic vs. foreign emission sources on the related health impacts is investigated using the emission perturbation scenarios. A global reduction of anthropogenic emissions by 20 % decreases the health impacts by 17 %, while the reduction of foreign emissions decreases the health impacts by less than 1 %. The decrease of emissions within the source region decreases the health impacts by 16 %. These results show that the largest impacts of reducing emissions with respect to the premature deaths come from emissions within the source region, while foreign sources contribute to much less improvement in avoiding adverse impacts of air pollution.

## 5 Outlook

Currently, health assessments of airborne particles are carried out under the assumption that all fine-fraction particles affect health to a similar degree, independent of origin, age and chemical composition of the particles. A 2013 report from WHO concludes that the cardiovascular effects of ambient PM_2.5_ are greatly influenced, if not dominated, by their transition metal contents (WHO, 2013b). It is known that trace metals and traffic markers are highly associated with daily mortality (Lippmann, 2014). Even low concentrations of trace metals can be influential for health-related responses.

Regarding ambient concentrations of PM and the ERFs, there is a rich set of studies providing information on total PM mass. However, only few studies focus on individual particulate species, mainly black carbon and carbonaceous particles. In addition to PM, studies on human populations have not been able to isolate potential effects of NO_2_, because of its complex link to PM and O_3_. The WHO REVIHAAP review from 2013 concludes that health assessments based on PM_2.5_ ERFs will be most inclusive (WHO, 2013b). In addition, the ERFs are based on urban background measurements, introducing uncertainties regarding non-urban areas or high pollution areas, e.g., street canyons. Current state-of-the-art health impact estimates, in particular on regional to global scales, assume a correlation with exposure to outdoor air pollution, while in reality, exposure is dynamic and depends on the behavior of the individual. In addition, differences in age groups, gender, ethnicity and behavior should be considered in the future studies. There are also uncertainties originating from the representations of the aerosols in the atmospheric models used in the calculation of pollutant concentrations as well as the emissions. Further developments in the aerosol modules, such as the representation of organic aerosols and windblown and suspended dust, are need in order to achieve mass closure of PM to get robust estimates of health impacts. In addition, new findings show that O_3_ has also chronic health impacts in addition to its acute impacts (WHO, 2013a; Turner, 2016).

Due to above reasons, there is a large knowledge gap regarding the health impacts of particles. There are a number of ongoing projects trying to identify the health impacts from individual particle components and produce individual ERFs for these components. NordicWelfAir project (http://projects.au.dk/nordicwelfair/) aims to investigate the potential causal impact of individual chemical air pollutants as well as mixtures of air pollutants on health outcomes. In pursuing this aim, the project uses the unique Nordic population-based registers, allowing linkage between historical residential address, air pollutants over decades and later health outcomes. By linking the exposure to health outcomes, new exposure–response relationships can be determined on health effects for different population groups (e.g., age, education, ethnicity, gender, lifestyle and working life vs. retirement conditions) related to air pollution for the individual chemical air pollutants. In addition, the high-resolution simulations conducted will enable us to have a better understanding of non-linearities between the emissions, health impacts and their economic valuation.

## Supplementary Material

Supp

## Figures and Tables

**Table 1:** Key features (meteorological/chemistry–transport models, emissions, horizontal and vertical grids) of the regional models participating to the AQMEII3 health impact study and the perturbation scenarios they performed.

Group code	Model	Emissions	Horizontal resolution	Vertical resolution	Gas phase	Aerosol model	Europe				North America			

							BASE	GLO	NAM	EUR	BASE	GLO	EAS	NAM
DE1	COSMO-CLM/CMAQ	HTAP	24 km × 24 km	30 layers, 50 hPa	CB5-TUCL	3 modes	x	x	x	x	x	x	x	x
DK1	WRF/DEHM	HTAP	50 km × 50 km	29 layers, 100 hPa	Brandt et al. (2012)	2 modes	x	x	x	x	x	x	x	x
ES1	WRF/CHEM	MACC	23 km × 23 km	33 layers, 50 hPa	RADM2	3 modes, MADE/SORGAM	x		x					
FI1	ECMWF/SILAM	MACC	0.25° × 0.25°	12 layers, 13 km	CB4	1–5 bins, VBS	x	x	x	x				
FRES1	ECMWF/CHIMERE	HTAP	0.25° × 0.25°	9 layers, 50 hPa	MELCHIOR2	8 bins	x	x	x	x				
IT1	WRF/CHEM	MACC	23 km × 23 km	33 layers, 50 hPa	RACM-ESRL	3 modes, MADE/VBS	x	x		x				
IT2	WRF/CAMx	MACC	23 km × 23 km	14 layers, 8 km	CB5	3 modes	x	x						
NL1	LOTOS/EUROS	MACC	0.50° × 0.25°	4 layers, 3.5 km	CB4	2 modes, VBS	x							
TR1	WRF/CMAQ	MACC	30 km × 30 km	24 layers, 10 hPa	CB5	3 modes	x	x	x					
UK1	WRF/CMAQ	MACC	15 km × 15 km	23 layers, 100 hPa	CB5-TUCL	3 modes	x	x	x	x				
UK2	WRF/CMAQ	HTAP	30 km × 30 km	23 layers, 100 hPa	CB5-TUCL	3 modes	x	x						
UK3	WRF/CMAQ	MACC	18 km × 18 km	35 layers, 16 km	CB5	3 modes	x	x	x					
US3	WRF/CMAQ	SMOKE	12 km × 12 km	35 layers, 50 hPa	CB5-TUCL	3 modes					x	x	x	x

**Table 2:** Exposure–response functions, the concentrations metrics and economic valuations used in the EVA model. “EU27” are the member states of the European Union between 2007 and 2013.

Health effects (compounds)	Exposure–response coefficient	Valuation, EUR_2013_

	(*α*)	(EU27 & NA)
Morbidity		

Chronic bronchitis^1^, CB (PM)	8.2E-5 cases μg^−1^ m^−3^ (adults)	38 578 per case
Restricted activity days^2^, RAD (PM)	= 8.4 E-4 days μg^−1^ m^−3^ (adults)	98 per day
	−3.46E-5 days μg^−1^ m^−3^ (adults)	
	−2.47E-4 days μg^−1^ m^−3^(adults > 65)	
	−8.42E-5 days μg^−1^ m^−3^ (adults)	

Congestive heart failure^3^, CHF (PM)	3.09E-5 cases μg^−1^ m^−3^	10 998 per case
Congestive heart failure^3^, CHF (CO)	5.64E-7 cases μg^−1^ m^−3^	
Lung cancer^4^, LC (PM)	1.26E-5 cases μg^−1^ m^−3^	16 022 per case

Hospital admissions		

Respiratory^5^, RHA (PM)	3.46E-6 cases μg^−1^ m^−3^	5315 per case
Respiratory^5^, RHA (SO_2_)	2.04E-6 cases μg^−1^ m^−3^	
Cerebrovascular^6^, CHA (PM)	8.42E-6 cases μg^−1^ m^−3^	6734 per case

Asthma children (7.6 % < 16 years)		

Bronchodilator use^7^, BUC (PM)	1.29E-1 cases μg^−1^ m^−3^	16 per case
Cough^8^, COUC (PM)	4.46E-1 days μg^−1^ m^−3^	30 per day
Lower respiratory symptoms^7^, LRSA (PM)	1.72E-1 days μg^−1^ m^−3^	9 per day

Asthma adults (5.9 % > 15 years)		

Bronchodilator use^9^, BUA (PM)	2.72E-1 cases μg^−1^ m^−3^	16 per case
Cough^9^, COUA (PM)	2.8E-1 days μg^−1^ m^−3^	30 per day
Lower respiratory symptoms^9^, LRSA (PM)	1.01E-1 days μg^−1^ m^−3^	9 per day

Mortality		

Acute mortality^10^,^11^ (SO_2_)	7.85E-6 cases μg^−1^ m^−3^	1 532 099 per case
Acute mortality^10^,^11^ (O_3_)	3.27E-6 × SOMO35 cases μg^−1^ m^−3^	
Chronic mortality^4^,^12^, YOLL (PM)	1.138E-3 YOLL μg^−1^ m^−3^(> 30 years)	57 510 per YOLL
Infant mortality^13^, IM (PM)	6.68E-6 cases μg^−1^ m^−3^ (> 9 months)	2 298 148 per case

1 Abbey et al. (1995).

2 Ostro (1987).

3 Schwartz and Morris (1995).

4 Pope et al. (2002).

5 Dab et al. (1996).

6 Wordley et al. (1997).

7 Roemer et al. (1993).

8 Pope and Dockerey (1992).

9 Dusseldorp et al. (1995).

10 Anderson et al. (1996).

11 Touloumi et al. (1996).

12 Pope et al. (1995).

13 Woodruff et al. (1997).

**Table 3:** Health impacts calculated by the mean of individual model estimates (denoted as MM_mi_) and the standard deviation, multi-model mean ensemble without error reduction (MM_m_) and the optimal ensemble (MM_opt_) in Europe and the US. See Table 2 for the definitions of health impacts. PD stands for premature deaths. All health impacts are in units of number of cases multiplied by 1000, except for infant mortality (IM), which reports directly the number of cases.

	EU			NA		

	MM_mi_	MM_m_	MM_opt_	MM_mi_	MM_m_	MM_opt_
CB	360 ± 89	360	468	142 ± 74	142	125
RAD	368 266 ± 90 670	368 245	478 073	145 337 ± 75 250	145 337	127 921
RHA	23 ± 5	23	28	10 ± 4	8	7
CHA	46 ± 11	46	60	19 ± 10	19	16
CHF	31 ± 6	31	38	13 ± 6	9	8
LC	55 ± 14	55	72	22 ± 11	22	19
BDUC	10 766 ± 2650	10 766	13 976	4566 ± 2383	4566	4019
BDUA	70 492 ± 17 400	70 489	91 511	27 819 ± 14 400	27 819	24 485
COUC	37 198 ± 9160	37 196	48 289	15 776 ± 8230	15 776	13 886
COUA	72 566 ± 17 900	72 562	94 203	28 637 ± 14 830	28 637	25 206
LRSC	14 355 ± 3530	14 354	18 635	6088 ± 3180	6088	5359
LRSA	26 175 ± 6400	26 174	33 980	10 330 ± 5350	10 330	9092
AYOLL	26 ± 13	23	20	25 ± 7	9	9
YOLL	4111 ± 1010	4111	5337	1481 ± 762	1481	1304
PD	414 ± 98	410	524	165 ± 76	149	133
IM	403 ± 99	403	524	143 ± 75	143.3667	126.1

**Table 4:** External costs (in million EUR) related to the health impacts of air pollution as calculated by the individual models over Europe and the United States.

Models	CO	SO_2_	O_3_	PM_2.5_	Total
Europe					

DE1	70	19 000	22 000	155 000	196 000
DK1	80	13 000	24 000	237 000	274 000
ES1	70	8000	6000	339 000	353 000
FI1	90	18 000	5000	335 000	358 000
FRES1	90	15 000	13 000	305 000	333 000
IT1	80	17 000	21 000	413 000	451 000
IT2	70	11 000	6000	253 000	270 000
NL1	70	12 000	18 000	215 000	245 000
TR1	110	30 000	35 000	376 000	441 000
UK1	80	28 000	25 000	280 000	333 000
UK2	80	34 000	27 000	340 000	401 000
UK3	80	47 000	25 000	279 000	351 000

Mean	81	21 000	19 000	294 000	334 000
Median	80	17 500	21 500	292 500	342 000

United States					

DE1	30	9000	21 000	46 000	76 000
DK1	55	11 000	39 000	123 000	172 000
US3	60	14 000	22 000	155 000	191 000

Mean	50	11 500	27 000	108 000	146 000
Median	55	11 000	22 000	123 000	172 000

**Table 5:** Annual average RMSEs of the multi-model ensemble mean (MM_m_) and of the optimal reduced ensemble mean (MM_opt_) for the health-impact-related species. Units are in ppb for the gaseous species and μg m^−3^ for PM_2.5_.

	O_3_		CO		SO_2_		PM_2.5_	

	MM_m_	MM_opt_	MM_m_	MM_opt_	MM_m_	MM_opt_	MM_m_	MM_opt_
Europe								

Winter	10.3	8.6	502.4	490.3	6.3	5.6	22.5	20.7
Spring	12.4	9.6	247.1	239.5	4.6	3.1	9.9	7.8
Summer	13.4	10.7	197.4	188.0	3.9	2.3	8.2	5.7
Autumn	10.7	8.8	314.5	305.5	4.6	3.1	11.0	8.7

Annual	11.7	9.4	315.3	305.8	4.8	3.5	12.9	10.7

North America								

Winter	10.9	10.4	356.7	328.1	5.7	5.5	8.3	8.1
Spring	12.0	11.4	288.7	270.2	5.4	5.1	7.2	6.6
Summer	15.1	13.0	258.3	238.7	5.4	5.0	9.7	8.8
Autumn	12.8	11.6	330.6	307.6	5.8	5.3	7.8	7.2

Annual	12.7	11.6	308.6	286.1	5.6	5.2	8.2	7.7

**Table 6:** Impact of the emission reduction scenarios on avoided premature deaths (ΔPD) and corresponding change in external cost as calculated by the multi-model mean over Europe and the United States.

	Receptor			
	
	Europe		United States	
	
Source	ΔPD	Δ Total cost (billion EUR)	ΔPD	Δ Total cost (billion EUR)
GLO	−54 000 ± 18 000	−56 ± 18	−27 500 ± 14 000	−24 ± 10
NAM	−940 ± 1100	−1.4 ± 0.4	−25 000 ± 12 000	−21 ± 9
EUR	−47 000 ± 24 000	−7 ± 16	–	–
EAS	–	–	−1900 ± 2200	−2.5 ± 3
