# Supplementary material for: Assessment and economic valuation of air pollution impacts on human health over Europe and the United States as calculated by a multi-model ensemble in the framework of AQMEII3
Source: Atmos Chem Phys. Author manuscript; Available in PMC 2018 Aug 1. (PMC6070159; doi:10.5194/acp-18-5967-2018)
Supplement: Supp [file NIHMS982660-supplement-Supp.pdf]

Supplement of Atmos. Chem. Phys., 18, 5967–5989, 2018
https://doi.org/10.5194/acp-18-5967-2018-supplement
© Author(s) 2018. This work is distributed under
the Creative Commons Attribution 4.0 License.

Atmospheric Chemistry and Physics
Open Access

Supplement of

Assessment and economic valuation of air pollution impacts on human health over Europe and the United States as calculated by a multi-model ensemble in the framework of AQMEII3

Ulas Im et al.

Correspondence to: Ulas Im (ulas@envs.au.dk)

The copyright of individual parts of the supplement might differ from the CC BY 4.0 License.

Model Features

The FMI (Finnish Meteorological Institute: FI1) used the SILAM v5.4 (Sofiev et al., 2015) model, driven by the meteorological input extracted from the ECMWF operational archives. The simulation included sea salt emissions as in Sofiev et al. (2011) (but not from the boundaries), biogenic VOCs (volatile organic compounds) emissions as in Poupkou et al. (2010), and wildland fire emissions as in Soares et al. (2015). The windblown dust is only included from the lateral boundary conditions. The volatility distribution of anthropogenic organic carbon (OC) was taken from Shrivastava et al. (2011). The gasphase chemistry was simulated with CBM-IV, with reaction rates updated according to the recommendations of International Union of Pure and Applied Chemistry (IUPAC) (http: //iupac.pole-ether.fr) and the NASA Jet Propulsion Laboratory (JPL) (http://jpldataeval.jpl.nasa.gov). The secondary inorganic aerosol formation was computed with the updated DMAT scheme (Sofiev, 2000) and secondary organic aerosol formation with the volatility basis set (VBS; Ahmadov et al., 2012). Pressure- and latitude-dependent photolysis rates of the FinROSE model (Damski et al., 2007) are used and reduced proportionally to cloud cover below the clouds down to half the original value at full cloud cover. The SILAM model does not account for extra plume rise in addition to that prescribed by the emission profiles. A known deficiency of the SILAM version used in this study is the overestimation of ozone dry deposition.

TNO (the Netherlands Organisation for Applied Scientific Research- NL1) used the LOTOS-EUROS modeling system (Schaap et al., 2008, Sauter et al., 2012). The meteorological inputs were extracted from the ECMWF operational archives. For biogenic emissions the approach as described in Beltman et al. (2013) was used. Gas-phase chemistry is based on CBM-IV (modified reaction rates; see Sauter et al., 2012), secondary inorganic aerosol (SIA) formation on ISORROPIA II (Fountoukis and Nenes, 2009), and for semivolatile species the VBS approach was used (Donahue et al., 2006; Bergström et al., 2012), with 100% of the emitted OC mass in the four lowest volatility classes that are predominantly solid and an additional 150% in the five higher volatility bins. Modeled terpene emissions were reduced by 50% to limit their contribution to SOA (secondary organic aerosol) formation, which was found to be too high otherwise (Bergström et al., 2012). No NOx emissions from soil were taken into account. The model includes pH-dependent conversion rates for SO2 (Banzhaf et al., 2012), while only below-cloud scavenging is used for wet deposition. Mineral dust emissions were calculated online, including emissions from road resuspension and agricultural activities, according to Schaap et al. (2009). For sea spray the parameterizations by Monahan et al. (1986) and Martensson et al. (2003) were used. Photolysis rates are based on clear-sky photolysis rate by Roeth's flux algorithm (function of solar zenith angle; Poppe et al., 1996) and multiplied by an attenuation factor in case of clouds. The LOTOS-EUROS model does not account for extra plume rise in addition to that prescribed by the emission profiles. A specific feature of LOTOS-EUROS is that it only covers the lower 3.5 km of the atmosphere, with a static 25m surface layer, a dynamic mixing layer and two dynamic reservoir layers. This makes the model relatively fast in terms of computation time but has implications for the vertical mixing of species for instances where the mixing layer rapidly changes in height.

The INERIS and CIEMAT institutes jointly (FRES1) applied the ECMWF-CHIMERE system. CHIMERE (version CHIMERE 2013) was run with meteorology provided by ECMWF IFS. Biogenic VOC emissions from vegetation and soil NO emissions were calculated with the MEGAN model (version 2.04; Guenther et al., 2006, 2012). Sea salt emissions inside the domain were calculated according to Monahan (1986). The windblown dust is only included from the lateral boundary conditions. CHIMERE uses the MELCHIOR2 chemical mechanism (Lattuati, 1997), and ammonium nitrate equilibrium was calculated with ISORROPIA (Nenes et al., 1999). Dry deposition is based on the resistance approach (Emberson 2000a, b) and both in-cloud and sub-cloud scavenging were considered for wet deposition.

University of L'Aquila (IT1) used the Weather Research and Forecasting model with Chemistry (WRF-Chem) Version 3.6, which has been modified to include the new chemistry option implemented by Tuccella et al. (2015) that includes a better representation of the secondary organic aerosol mass in the simulation of direct and indirect aerosol effects, calculated as in Ahmadov et al. (2012). Here only direct effects were included in the simulation, for computational expediency. The model uses the RACM-ESRL gas-phase chemical mechanism (Kim et al., 2009), an updated version of the Regional Atmospheric Chemistry Mechanism (RACM; Stockwell et al., 1997). The inorganic aerosols are treated with the Modal Aerosol Dynamics model for Europe (MADE; Ackermann et al., 1998). The parameterization for SOA production is based on the volatility basis set (VBS) approach. The aerosol direct and semi-direct effects are taken into account following Fast et al. (2006). Cloud chemistry in the convective updraft is modeled using the scheme of Walcek and Taylor (1986), while the aqueous-phase oxidation of SO2 by H2O2 in the grid-resolved clouds is parameterized with the scheme used in GOCART (Goddard Chemistry Aerosol Radiation and Transport).Wet deposition from convective and resolved precipitation is included following Grell and Freitas (2014). The photolysis frequencies are calculated with the Fast-J scheme (Fast et al., 2006). Dry deposition and photolysis schemes were modified to take into account the effects of the soil snow coverage following Ahmadov et al. (2015).

University of Murcia (ES1) used the WRFChem2 model. The following physics options were applied for the simulations: rapid radiative transfer method for global (RRTMG) long-wave and shortwave radiation scheme, Lin microphysics (Lin et al., 1993), the Yonsei University (YSU) PBL scheme (Hong et al., 2006), the NOAH land-surface model, and the updated version of the Grell–Devenyi scheme (Grell and Devenyi, 2002) with radiative feedback. Chemical options include RADM2 chemical mechanism (Stockwell et al., 1990), MADE/SORGAM aerosol module (Schell et al., 2001) including some aqueous reactions, and Fast-J photolysis scheme. The modeling domain covers Europe and a portion of North Africa.

RSE (IT2) used WRF-CAMx version 6.10 (Environ, 2014) with Carbon Bond 2005 (CB05) gas-phase chemistry (Yarwood et al., 2005) and the coarse–fine (CF) aerosol module. Input meteorological data were generated by WRF model version 3.4.1 (Skamarock et al., 2008), driven by ECMWF analysis fields. Grid nudging of wind speed, temperature, and water vapor mixing ratio was employed within the PBL, with a nudging coefficient of 0.0003 s^{-1}. WRF-Chem was adopted to predict GOCART dust emissions (Ginoux et al., 2001) along with the meteorology.The WRF-CAMx preprocessor (version 4.2; Environ, 2014) was used to create

CAMx ready input files, collapsing the 33 vertical layers used by WRF into 14 layers in CAMx but keeping the layers up to 230m above ground level identical. Biogenic VOC emissions were computed by applying the MEGAN emission model v2.04. Sea salt emissions were computed using published algorithms (de Leeuw et al., 2000; Gong, 2003).

Aarhus University (DE1) applied the WRF-DEHM modeling system over EU and NA. The DEHM model used anthropogenic emissions from the EDGAR-HTAP database and biogenic emissions were calculated using the MEGAN model. The gas-phase chemistry module includes 58 chemical species, 9 primary particles, and 122 chemical reactions (Brandt et al., 2012). Secondary organic aerosols (SOA) were calculated following the two-product approach assuming that hydrocarbons undergo oxidation through O3, OH, and NO3 and for only two semi-volatile gas products (Zare et al., 2014). However, the module is simple because it does not include aging processes and further reactions in the gas and particulate phases (Zare et al., 2014).

Istanbul Technical University (TR1) used the WRF-CMAQ. The Meteorology-Chemistry Interface Processor (MCIP) version 3.6 (Otte and Pleim, 2010) was used to process WRF output for CMAQ. The MEGAN v2.1 (Guenther et al., 2012) model was used to calculate the biogenic VOC emissions from vegetation, using surface temperature and radiation from MCIP output. CMAQ v4.7.1 (Foley et al., 2010) was configured with the CB05 chemical mechanism and the AERO5 module (Foley et al., 2010) for the simulation of gas-phase chemistry and aerosol and aqueous chemistry, respectively.

Ricardo Energy & Environment (Ricardo E&E: UK2) used the WRF-CMAQ system. It was configured using WRF v3.5.1 and CMAQ v5.0.2. The CMAQ model adopted the CB05-TUCL chemical mechanism (Whitten et al., 2010; Sarwar et al., 2011) and the AERO6 threemode aerosol module (Appel et al., 2013). MCIP version 4.2 was used to process WRF output for CMAQ. The MEGAN v2.0.4 model was used to calculate the biogenic VOC emissions from vegetation, using surface temperature and radiation from MCIP output.

University of Hertfordshire (UK1) used the WRF-CMAQ modeling system utilized the uncoupled version of the WRF v3.4.1 model and CMAQ v5.0.2. The results from WRF simulations were preprocessed for CMAQ using MCIP version 3.6 (Otte et al., 2005). In the CMAQ model, the gas-phase chemical mechanism was based on Carbon Bond chemical mechanism version 5 (Foley et al., 2010) with updated toluene and chlorine chemistry (CB05-TUCL), and the aerosol chemical reaction was treated with AERO6 module. The biogenic emissions were derived from MEGAN.

Kings College (UK3) used the WRF-CMAQ model, using CMAQ v5.0.2 (Byun and Schere, 2006), with CB05 chemical mechanism that included aqueous and aerosol chemistry. The CMAQ model is driven by meteorological fields from WRF v3.4.1. The anthropogenic emissions for most of the model domain are from MACC and the missing information was filled with the emissions provided by EDGAR/HTAP. The biogenic emissions were estimated using the BEIS3 model. The dust and sea salt (Gantt et al., 2015) emissions were generated using CMAQ inline modules.

HZG (DE1) used the COSMO-CLM meteorological model to drive the CMAQ model. For AQMEII3, CMAQ version 5.0.1 was used, with the CB05-TUCL scheme and the multipollutant aerosol module AERO6. CMAQ was run using the optional in-line calculation of dry deposition velocities. Wet deposition processes include in-cloud and sub-cloud scavenging processes. All atmospheric parameters were taken from regional atmospheric simulations with the COSMOCLM (CCLM) mesoscale meteorological model (version 4.8) for the year 2010 (Geyer, 2014) using National Centers for Environmental Prediction (NCEP) forcing data employing a spectral nudging method for large-scale effects (Kalnay et al., 1996). CCLM is the climate version of the regionalscale meteorological community model COSMO (Schaettler et al., 2008). CCLM uses the TERRA-ML land surface model (Schrodin and Heise, 2001), a turbulent kinetic energy (TKE) closure scheme for the PBL (Doms et al., 2011), cloud microphysics after Seifert and Beheng (2001), the Tiedtke scheme (Tiedtke, 1989) for cumulus clouds, and a long-wave radiation scheme following Ritter and Geleyn (1992). The meteorological fields were processed afterwards to match the 2424 km2 CMAQ grid using the LM-MCIP preprocessor. The emission input for CCLM-CMAQ is based on the EDGAR HTAPv2 database, interpolated to the CMAQ model grid and aggregated following the SNAP emission sector nomenclature. Sector-specific hourly temporal profiles and speciation factors of PM and VOC species were applied by the SMOKE for Europe emissions model (Bieser et al., 2011a). The temporal profiles used were fixed monthly, weekly, and diurnal profiles. Biogenic emissions and NO emissions from soil were calculated using the BEIS3 model. Sea salt emissions were calculated in-line by CMAQ, including sulfate emissions based on an average sulfate content of 7.7 %. Finally, fixed vertical profiles were applied for each source sector (Bieser et al., 2011b).

US EPA (US3) used the WRF-CMAQ system over NA and was configured using WRF v3.4 and CMAQ v5.0.2 (Appel et al., 2013; see also Foley et al., 2010 and Byun and Schere, 2006). The options used in these WRF and CMAQ simulations are identical to those described in Hogrefe et al. (2015), except that the current simulations were performed in offline rather than two-way coupled mode. Temperature, wind speed, and water vapor mixing ratio were nudged above the PBL following the approach described in Gilliam et al. (2012). Soil temperature and moisture were nudged following Pleim and Xiu (2003) and Pleim and Gilliam (2009). The NO2 =NOx split applied during SMOKE emission processing varies for different categories. For many categories the assumed split is 90% NO= 10% NO2, but for mobile sources the split varies for different types of vehicles and different emission processes.

Model Evaluation

The models are evaluated against the surface observations using normalized mean bias (*NMB*), normalized mean gross error (*NMGE*), root mean square error (*RMSE*) and correlation coefficient (*r*).

$$NMB = \frac{\sum_{i=1}^{n} |Mi - Oi|}{\sum_{i=1}^{n} Oi} \qquad \text{Eq.1}$$

$$NMGE = \frac{\sum_{i=1}^{n} |Mi - Oi|}{\sum_{i=1}^{n} Oi} \quad \text{Eq.2}$$

$$RMSE = \sqrt{\frac{\sum_{i=1}^{n}(Mi - Oi)^2}{n}} \quad \text{Eq.3}$$

$$r = \frac{1}{(n-1)} \sum_{i=1}^{n} \left(\frac{Mi - \bar{M}}{\sigma_M}\right)\left(\frac{Oi - \bar{O}}{\sigma_O}\right) \quad \text{Eq.4}$$

where *M* and *O* denote model and observations, while *n* denotes number of pairs.

Sensitivity Analysis of EVA

The effect of pollution concentrations (EVA input) on health impacts (EVA output) is investigated by means of pairwise regression analysis. The input dataset consists of the simulated pollutant by each model, averaged over all stations. The averaging was performed at 9 discrete percentiles (1, 5, 10, 25, 50, 75, 90, 95, and 99). The output dataset consists of the 14 health impact indices for each model. The Pearson Correlation Coefficient (PCC) between the simulated pollutant levels and health impacts are presented in Fig. S1 (X axis: the 9 percentiles, Y axis: PCC) as boxplot. The spread at each percentile marks the variability of PCC between the different health impact indices (HII). For the particular input-output configuration, the results show that the $PM_{2.5}$ drives the variability of the HIIs, with PCC in the range 0.75-0.88 for all percentiles except the 1st (0.63). All correlations are statistically significant. For the other pollutants, the correlations are mostly statistically non-significant.

More quantitative measures of sensitivity are evaluated from the standardized regression coefficients (SRCs). Assume bj is the linear regression coefficient of the dependent input xj (O_3, $PM_{2.5}$, SO_2, CO) and the independent variable y (health indices) using a multiple linear regression model. If σxj and σy represent the standard deviations of the dependent input xj and the independent variable y respectively, the SRCs are defined as bjσxj/σy. The SRCs offer a measure of sensitivity that is multi-dimensionally averaged and hence represent a global measure of sensitivity. The effectiveness of the standardized regression coefficients is conditional on the value of the model coefficient of determination (R^2). The fit of the regression (Table S1) is high (0.76-0.96), implying that the linear regression model is able to represent the majority of the variation of the HII. This also means that at least 76% of the variation of the health indices is explained by sole variations in the pollutants (i.e. without interactions). If all pollutants take extreme low values, O_3 has the largest effect on health indices. Within the interquartile range of the pollutants, the most important contribution to the HIIs is from $PM_{2.5}$, followed by CO (with much smaller influence though). At the upper tail of the pollutant distribution, $PM_{2.5}$ is the most important factor, followed by O_3 and SO_2. Again, $PM_{2.5}$ drives the variability of the health indices also using multivariate analysis.

Table S1. Model evaluation over the European and North American domains (hourly for O_3, CO and SO_2 and daily means for $PM_{2.5}$). Units are % for *NMB* and *NMGE*, μg m^{-3} for all species for Europe and ppb for the gaseous species and μg m^{-3} for $PM_{2.5}$ in North America.

	O_3				CO				SO_2				$PM_{2.5}$			
Models	*r*	*NMB*	*NMGE*	*RMSE*	*r*	*NMB*	*NMGE*	*RMSE*	*r*	*NMB*	*NMGE*	*RMSE*	*r*	*NMB*	*NMGE*	*RMSE*
Europe																
DE1	0.73	9.87	4.59	13.50	0.80	-42.07	41.75	133.24	0.77	4.34	21.07	1.33	0.88	-63.08	128.10	11.95
DK1	0.88	6.71	2.59	9.99	0.74	-41.67	43.10	135.84	0.85	-47.24	56.49	1.54	0.86	-45.69	56.82	9.65
ES1	0.79	-15.16	6.59	14.21	0.59	-46.27	55.42	147.82	0.78	-68.13	182.02	2.15	0.23	-30.84	44.68	9.66
FI1	0.84	-35.87	24.00	23.58	0.85	-26.75	15.78	92.11	0.82	-20.49	17.26	1.05	0.58	-26.98	29.18	8.02
FRES1	0.78	-9.65	4.79	12.51	0.82	-39.19	34.10	123.37	0.74	-76.81	320.13	2.44	0.87	-36.16	32.25	7.88
IT1	0.90	4.20	2.45	9.60	0.82	-36.81	31.23	120.35	0.79	-29.78	28.19	1.26	0.78	-18.25	14.88	6.06
IT2	0.92	-14.26	4.46	11.76	0.77	-43.53	45.13	136.44	0.81	-54.78	87.14	1.77	0.11	-48.40	87.23	11.65
NL1	0.92	-5.06	2.01	8.30	0.69	-46.09	55.74	148.51	0.80	-51.92	83.39	1.79	0.76	-55.55	99.64	11.56
TR1	0.86	8.09	8.91	18.65	0.84	-20.11	9.40	74.24	0.43	2.28	24.06	1.40	0.60	-19.16	21.08	7.17
UK1	0.91	7.51	2.13	9.10	0.59	-41.56	44.88	138.72	0.73	-12.96	16.19	1.06	0.78	-40.32	44.67	8.97
UK2	0.83	-2.75	4.17	12.10	0.64	-42.63	45.23	138.00	0.72	20.46	17.82	1.31	0.77	-28.28	23.59	7.15
UK3	0.78	-1.01	4.04	12.01	0.80	-45.04	48.32	139.60	0.64	48.75	46.00	2.34	0.94	-43.82	42.44	8.48
MEAN	0.84	-3.95	5.89	12.94	0.75	-39.31	39.17	127.35	0.74	-23.86	74.98	1.62	0.68	-38.04	52.05	9.02
MEDIAN	0.85	-1.88	4.32	12.06	0.78	-41.87	43.99	136.14	0.77	-25.14	37.10	1.47	0.77	-38.24	43.56	8.73
North America																
DE1	0.85	5.55	11.65	4.69	0.41	-40.68	40.71	92.20	0.45	-40.20	41.05	1.35	0.74	-62.65	62.65	6.97
DK1	0.72	21.75	23.80	10.33	0.47	-7.41	18.02	47.32	0.63	-42.36	43.47	1.35	0.64	-14.08	22.21	2.86
US3	0.88	-1.53	11.18	4.51	0.44	-3.89	19.89	51.42	0.52	-12.83	23.98	0.84	0.76	17.23	23.75	3.25
MEAN	0.82	8.59	15.54	6.51	0.44	-17.33	26.21	63.65	0.53	-31.80	36.17	1.18	0.71	-19.83	36.21	4.36
MEDIAN	0.85	5.55	11.65	4.69	0.44	-7.41	19.89	51.42	0.52	-40.20	41.05	1.35	0.74	-14.08	23.75	3.25

Table S2. Health impacts as calculated by the individual models over Europe and the United States (×10^3, except for IM). See Table 2 for the definitions of health impacts. PD stands for premature death (mortality).

Models	CB	RAD	RHA	CHA	CHF	LC	BUC	BUA	COUC	COUA	LRSC	LRSA	PD	IM
Europe														
DE1	191	194 776	13	24	19	29	5 694	37 284	19 674	38 380	7 592	13 844	232	213
DK1	290	296 611	17	37	26	44	8 671	56 776	29 960	58 446	11 562	21 082	336	325
ES1	415	424 229	23	53	34	64	12 402	81 205	42 851	83 593	16 536	30 153	456	465
FI1	411	420 220	25	53	35	63	12 285	80 437	42 445	82 803	16 380	29 868	457	460
FRES1	373	381 243	22	48	32	57	11 146	72 976	38 509	75 123	14 861	27 098	419	418
IT1	507	517 996	30	65	41	78	15 144	99 153	52 322	102 070	20 191	36 818	571	568
IT2	310	317 256	18	40	27	48	9 275	60 728	32 045	62 514	12 367	22 550	345	348
NL1	264	269 418	16	34	24	40	7 876	51 571	27 213	53 088	10 502	19 150	303	295
TR1	460	470 496	29	59	40	70	13 755	90 061	47 524	92 710	18 340	33 442	538	516
UK1	343	351 026	23	44	30	53	10 262	67 192	35 456	69 169	13 683	24 950	404	516
UK2	417	425 950	28	53	35	64	12 453	81 534	43 024	83 932	16 603	30 275	488	467
UK3	342	349 974	26	44	29	52	10 231	66 991	35 350	68 961	13 642	24 875	416	383
MEAN	360	368 266	23	46	31	55	10 766	70 492	37 198	72 566	14 355	26 175	414	414
MEDIAN	358	366 135	23	46	31	55	10 704	70 084	36 982	72 146	14 272	26 024	418	439
The United States														
DE1	61	62 305	5	8	7	9	1 946	11 926	6 722	12 277	2 594	4 428	80	61
DK1	161	164 681	10	21	15	25	5 148	31 522	17 787	32 449	6 864	11 705	191	161
US3	204	209 023	13	27	18	31	6 604	40 009	22 819	41 186	8 806	14 856	224	209
MEAN	142	145 337	10	19	13	22	4 566	27 819	15 776	28 637	6 088	10 330	165	143
MEDIAN	161	164 681	10	21	15	25	5 148	31 522	17 787	32 449	6 864	11 705	191	161

Table S3. Standardized regression coefficients and coefficient of model determination for the relations between concentration inputs and health impact indices in the EVA system.

Percentile	SRC_{O3}	$SRC_{PM2.5}$	SRC_{SO2}	SRC_{CO}	R^2
1st	-0.88	0.37	-0.37	0.32	0.84
5th	-0.15	0.76	0.38	-0.06	0.96
10th	-0.17	0.67	0.25	0.12	0.89
25th	-0.06	0.61	0.13	0.30	0.76
50th	0.12	0.74	-0.02	0.24	0.76
75th	0.27	0.81	-0.20	0.08	0.81
90th	0.38	0.76	-0.34	0.04	0.82
95th	0.45	0.72	-0.40	0.02	0.83
99th	0.56	0.69	-0.45	-0.01	0.89

Table S4. Health impacts as calculated by the individual models over Europe and the United States (×10^3, except for IM) in response to a 20% reduction of global anthropogenic emissions (GLO). See Table 2 for the definitions of health impacts.

Models	CB	RAD	RHA	CHA	CHF	LC	BUC	BUA	COUC	COUA	LRSC	LRSA	PD	IM*
Europe														
DE1	153	156 353	11	20	16	23	4 571	29 929	15 793	30 809	6 095	11 113	184	171
DK1	258	263 761	15	33	23	40	7 711	50 488	26 642	51 973	10 281	18 748	297	289
FI1	345	352 263	21	44	30	53	10 298	67 429	35 581	69 412	13 731	25 038	382	386
FRES1	305	312 159	18	39	27	47	9 126	59 753	31 531	61 510	12 168	22 187	342	342
IT1	414	423 452	24	53	34	63	12 380	81 056	42 772	83 440	16 506	30 098	467	464
IT2	267	273 062	16	34	23	41	7 983	52 269	27 581	53 806	10 644	19 409	296	299
TR1	378	386 412	24	48	33	58	11 297	73 965	39 030	76 141	15 062	27 465	459	423
UK1	282	287 915	19	36	25	43	8 417	55 112	29 082	56 733	11 223	20 464	5 370	315
UK2	333	340 662	22	43	28	51	9 959	65 208	34 409	67 126	13 279	24 213	390	373
MEAN	304	310 671	19	39	27	47	9 082	59 468	31 380	61 217	12 110	22 082	910	340
MEDIAN	305	312 159	19	39	27	47	9 126	59 753	31 531	61 510	12 168	22 187	382	342
The United States														
DE1	53	53735	3	7	4	8	1679	10286	5801	10588	2239	3819	62	52
DK1	132	135058	7	17	9	20	4221	25852	14585	26612	5628	9599	153	132
US3	168	171411	9	22	11	26	5414	32809	18707	33774	7219	12183	176	171
MEAN	117	120068	6	15	8	18	3772	22982	13031	23658	5029	8534	130	118
MEDIAN	132	135058	7	17	9	20	4221	25852	14585	26612	5628	9599	153	132

Table S5. Health impacts as calculated by the individual models over Europe and the United States (×10^3, except for IM) in response to a 20% reduction of North American anthropogenic emissions (NAM). See Table 2 for the definitions of health impacts.

Models	CB	RAD	RHA	CHA	CHF	LC	BUC	BUA	COUC	COUA	LRSC	LRSA	PD	IM*
Europe														
DE1	191	194 812	13	24	19	29	5 695	37 290	19 677	38 387	7 594	13 847	231	213
DK1	290	296 847	17	37	26	44	8 678	56 822	29 984	58 493	11 571	21 099	337	325
FI1	410	419 694	25	53	35	63	12 270	80 336	42 392	82 699	16 360	29 831	457	460
FRES1	372	379 831	22	48	32	57	11 104	72 706	38 366	74 844	14 806	26 997	418	416
TR1	460	469 841	29	59	40	70	13 736	89 936	47 458	92 581	18 314	33 395	536	515
UK1	342	349 960	23	44	30	52	10 231	66 989	35 349	68 959	13 641	24 874	403	383
MEAN	344	351 831	22	44	30	53	10 286	67 346	35 538	69 327	13 714	25 007	397	385
MEDIAN	357	364 896	22	46	31	55	10 668	69 847	36 857	71 902	14 224	25 936	410	400
The United States														
DE1	52	53 382	3	7	4	8	1 667	10 218	5 760	10 518	2 223	3 794	62	52
DK1	134	137 305	7	17	9	21	4 292	26 282	14 830	27 055	5 723	9 759	158	134
US3	169	172 620	9	22	11	26	5 454	33 041	18 845	34 013	7 273	12 269	178	172
MEAN	118	121 102	6	15	8	18	3 805	23 180	13 145	23 862	5 073	8 607	133	120
MEDIAN	134	137 305	7	17	9	21	4 292	26 282	14 830	27 055	5 723	9 759	158	134

Table S6. Health impacts as calculated by the individual models over Europe and the United States ($\times 10^3$, except for IM) in response to a 20% reduction of European (EUR) and East Asian (EAS) anthropogenic emissions. See Table 2 for the definitions of health impacts.

Models	CB	RAD	RHA	CHA	CHF	LC	BUC	BUA	COUC	COUA	LRSC	LRSA	PD	IM*
Europe														
DE1	154	157 444	11	20	16	24	4 603	30 138	15 903	31 024	6 137	11 191	187	173
DK1	270	275 740	16	35	24	41	8 061	52 781	27 852	54 333	10 748	19 599	312	302
FI1	346	353 923	21	44	30	53	10 347	67 747	35 749	69 740	13 796	25 156	384	388
FRES1	311	318 365	18	40	28	48	9 307	60 941	32 157	62 733	12 410	22 629	350	349
IT1	430	440 061	25	55	35	66	12 865	84 235	44 450	86 713	17 154	31 278	487	482
UK1	291	297 212	19	37	26	45	8 689	56 891	30 021	58 565	11 585	21 125	343	326
MEAN	300	307 124	18	39	27	46	8 979	58 789	31 022	60 518	11 972	21 830	344	336
MEDIAN	301	307 788	19	39	27	46	8 998	58 916	31 089	60 649	11 998	21 877	347	337
The United States														
DE1	61	62 470	3	8	4	9	1 951	11 958	6 740	12 309	2 601	4 440	74	61
DK1	160	163 776	9	21	11	25	5 119	31 349	17 687	32 271	6 826	11 641	180	160
US3	204	208 377	11	27	13	31	6 583	39 885	22 746	41 058	8 778	14 810	214	208
MEAN	142	144 875	8	18	9	22	4 551	27 730	15 724	28 546	6 068	10 297	156	143
MEDIAN	160	163 776	9	21	11	25	5 119	31 349	17 687	32 271	6 826	11 641	180	160

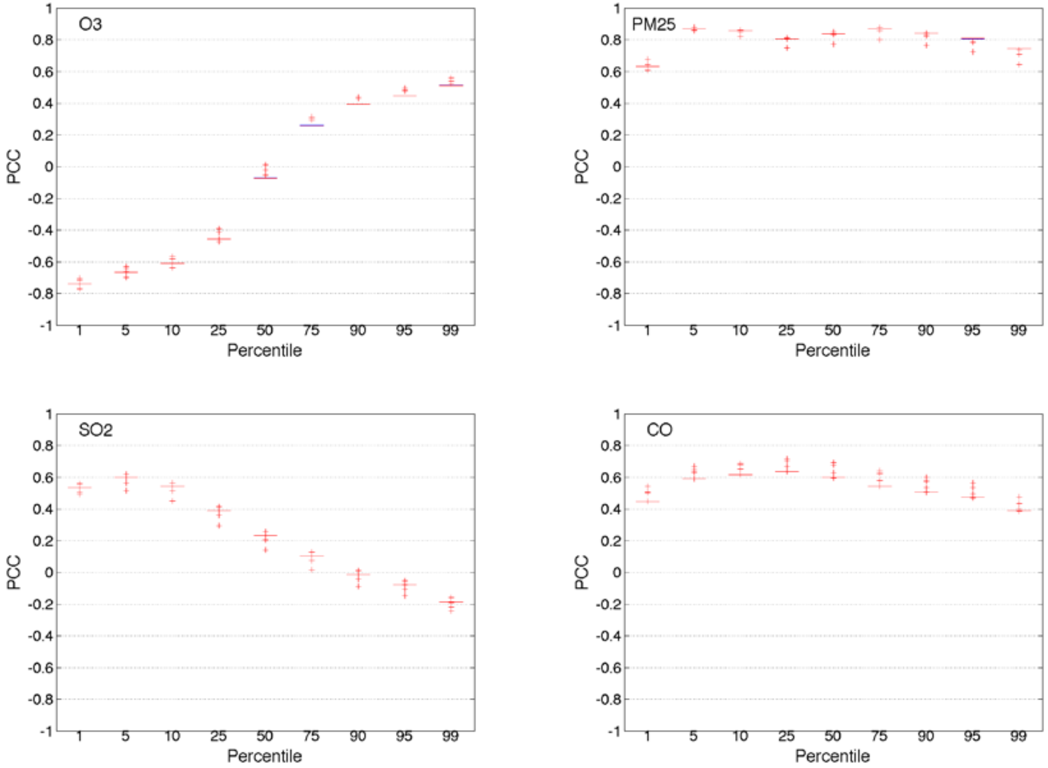

Fig. S1. The Pearson Correlation Coefficient (PCC) between the simulated pollutant levels at 9 discrete percentiles (1, 5, 10, 25, 50, 75, 90, 95, and 99) and health impacts.

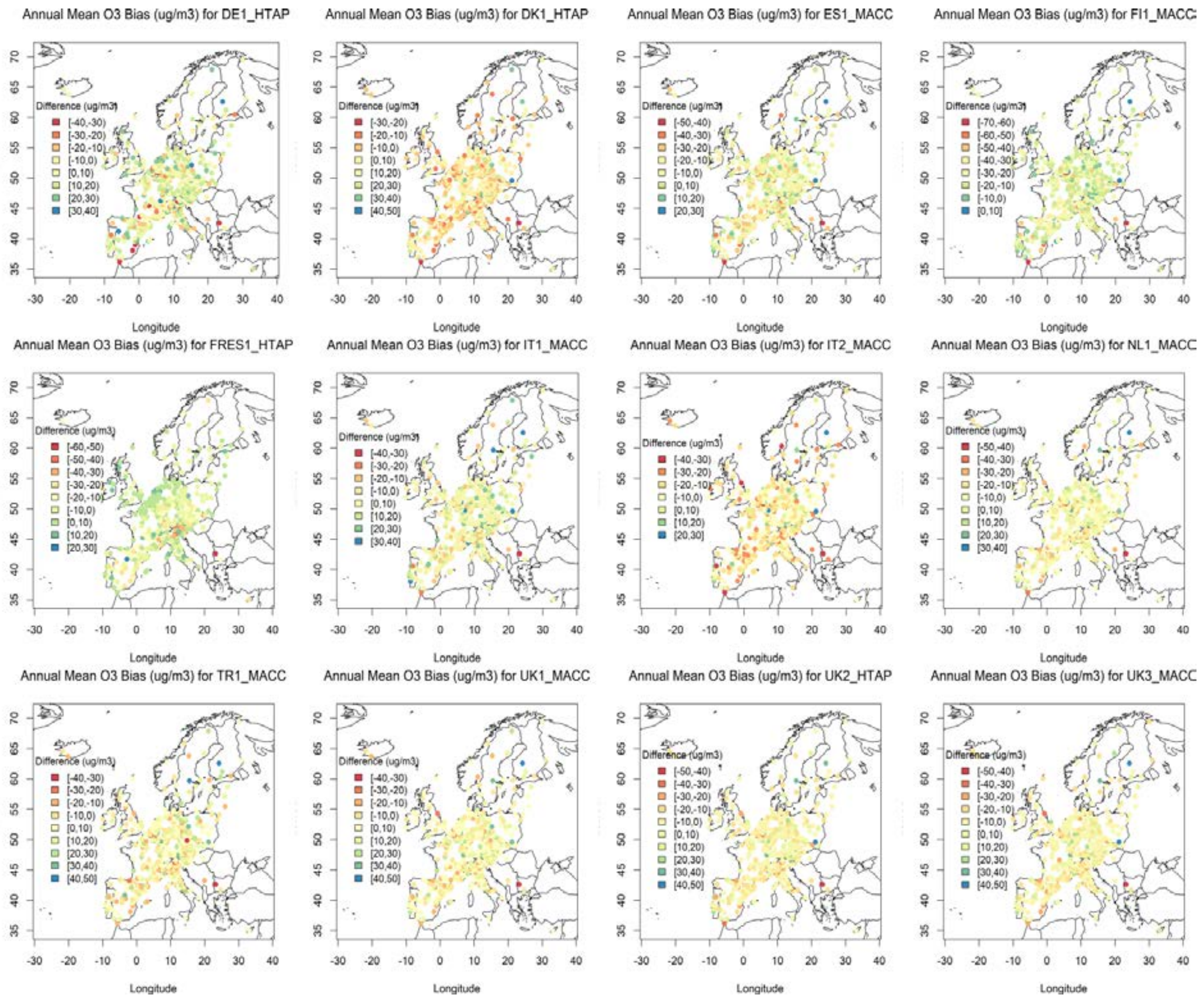

Fig. S2. Geographical distribution of bias (in units of μgm^{-3}) of simulated O_3 over Europe by individual models.

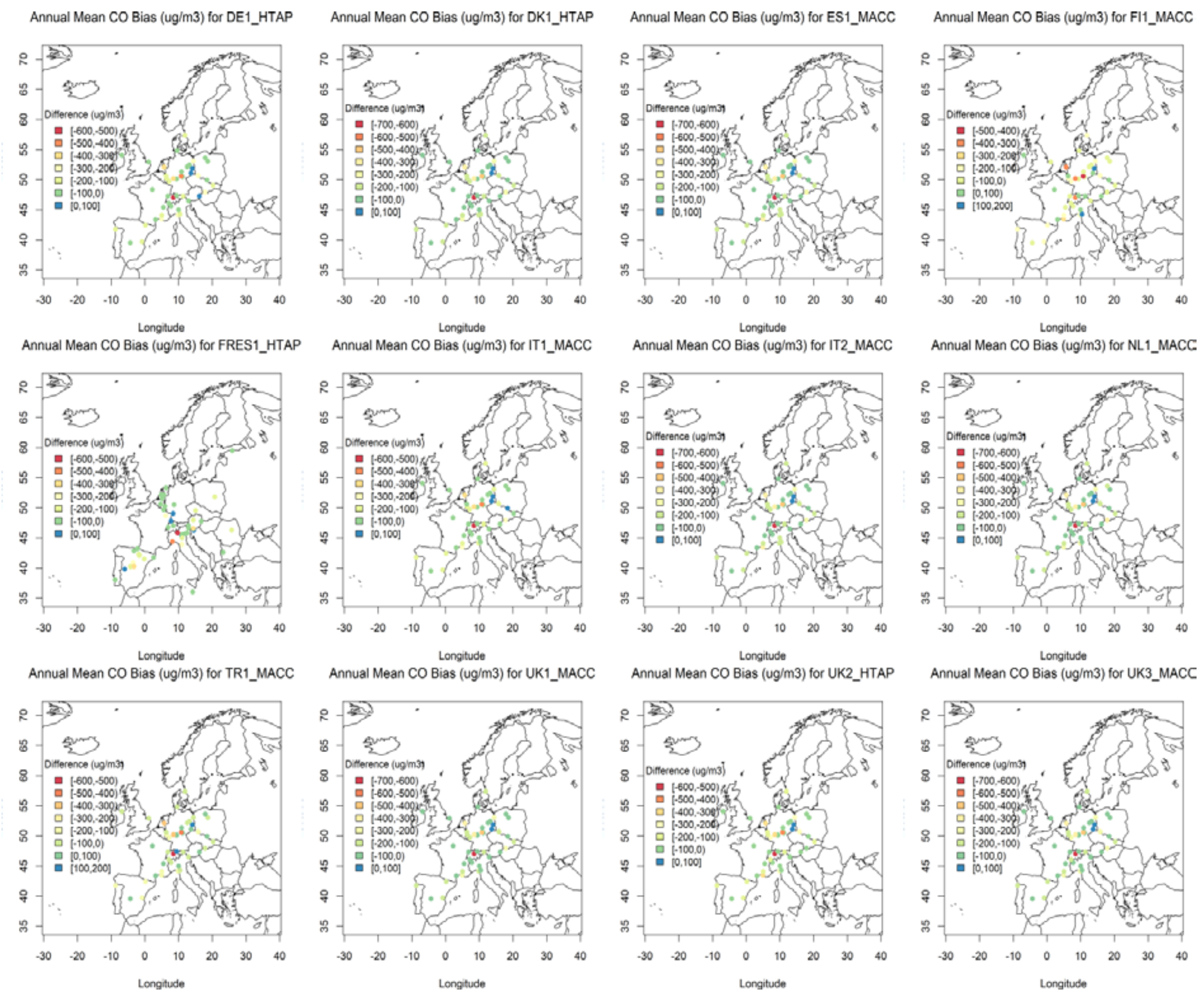

Fig. S3. Geographical distribution of bias (in units of μgm^{-3}) of simulated CO over Europe by individual models.

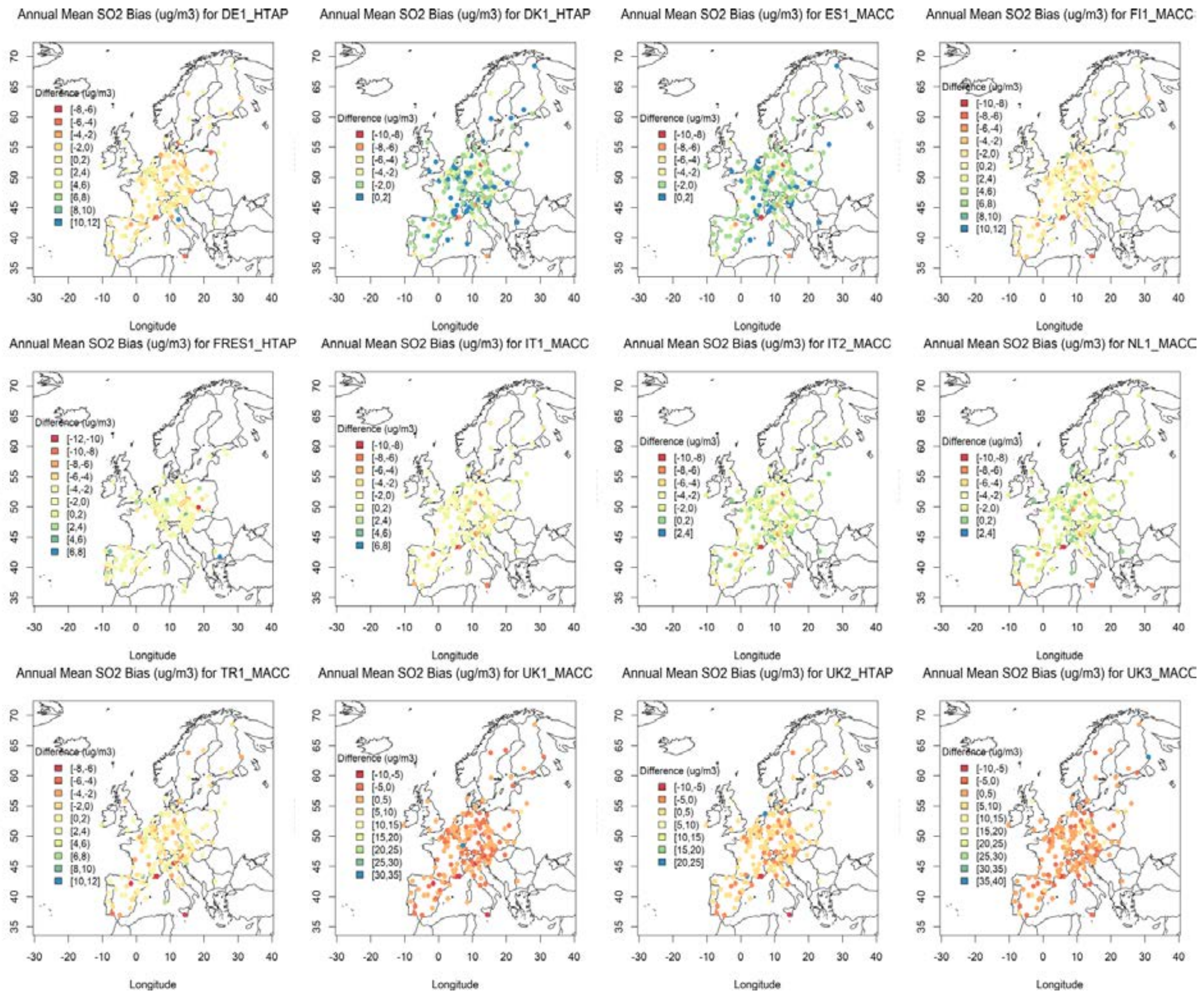

Fig. S4. Geographical distribution of bias (in units of μgm^{-3}) of simulated SO_2 over Europe by individual models.

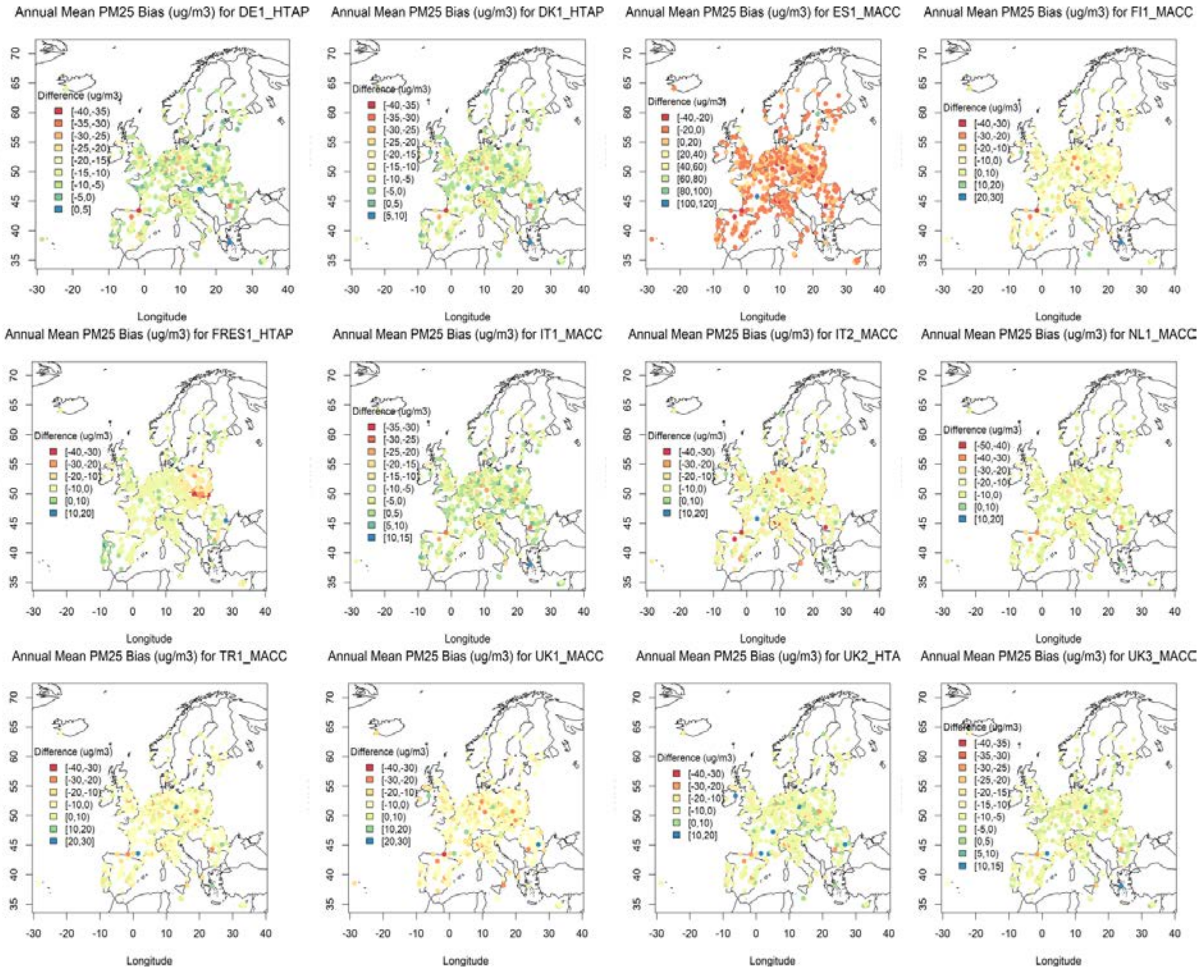

Fig. S5. Geographical distribution of bias (in units of μgm^{-3}) of simulated $PM_{2.5}$ over Europe by individual models.

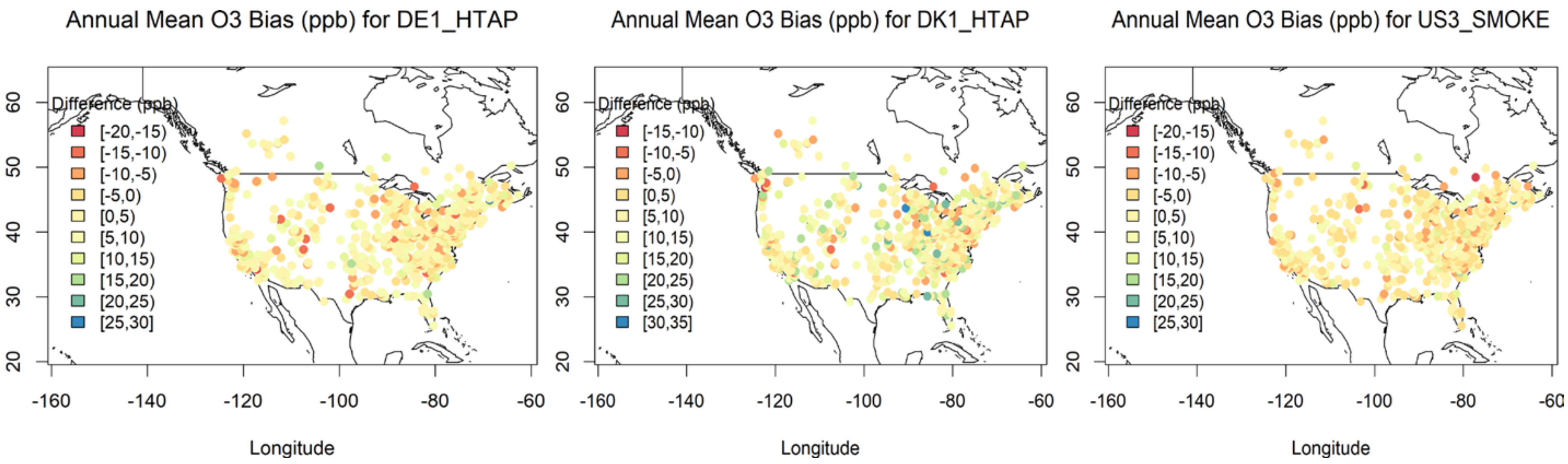

Fig. S6. Geographical distribution of bias (in units of ppb) of simulated O_3 over North America by individual models.

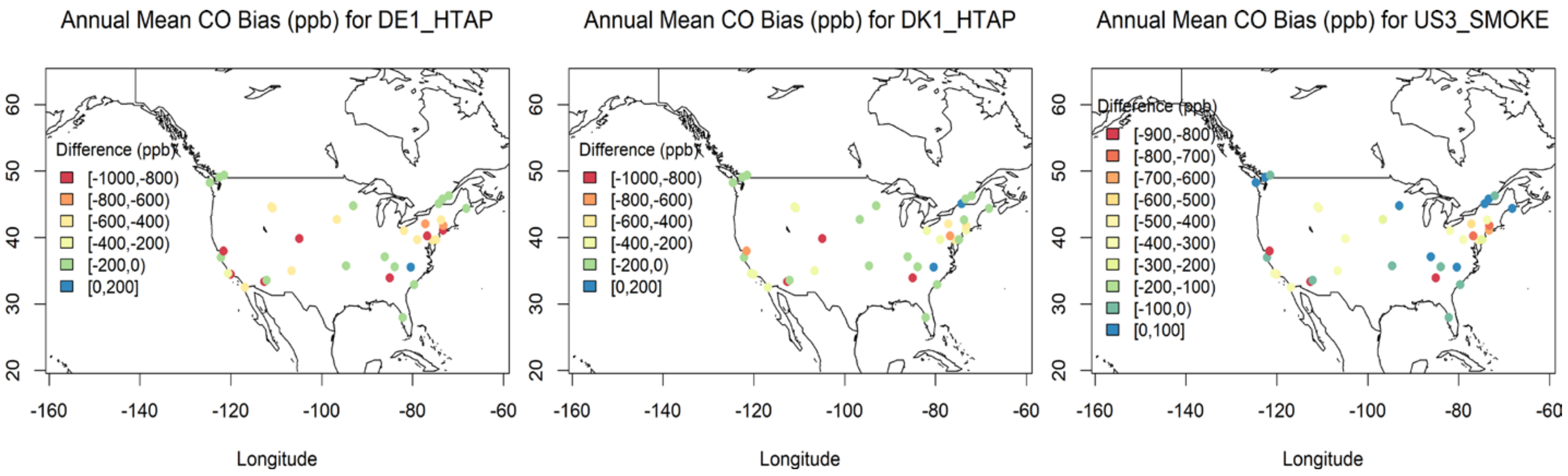

Fig. S7. Geographical distribution of bias (in units of ppb) of simulated CO over North America by individual models.

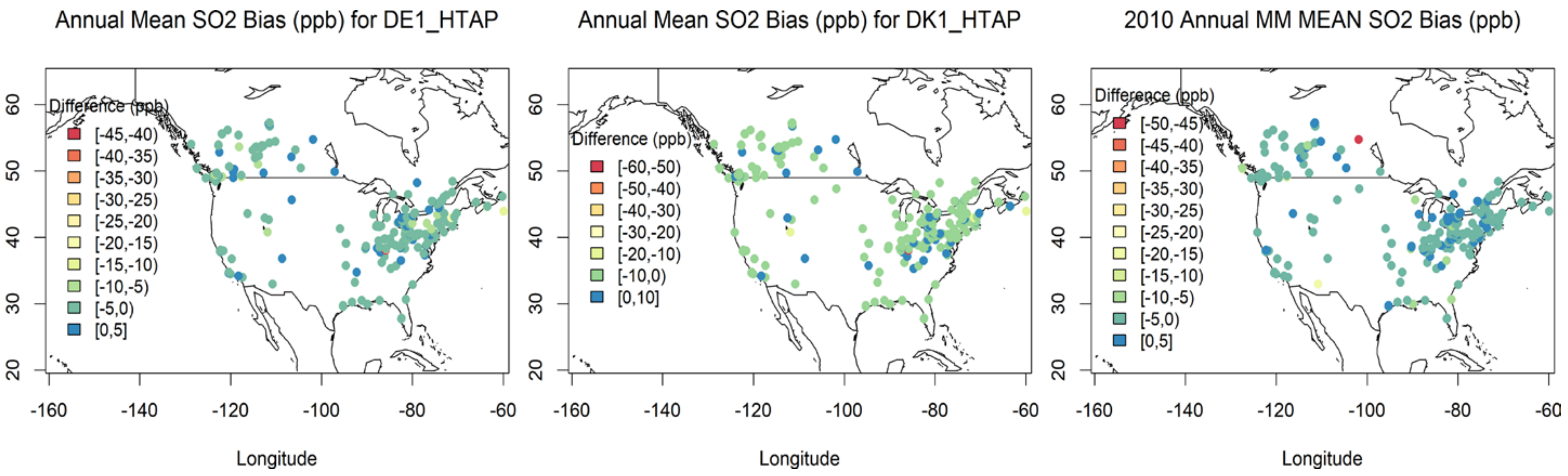

Fig. S8. Geographical distribution of bias (in units of ppb) of simulated SO_2 over North America by individual models.

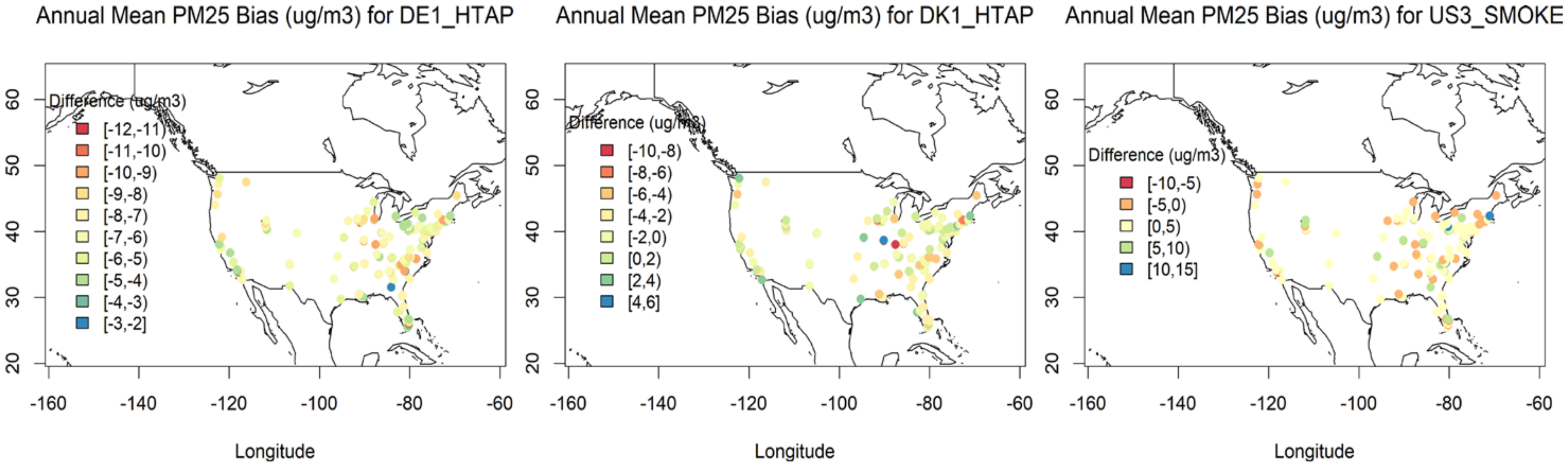

Fig. S9. Geographical distribution of bias (in units of ppb) of simulated $PM_{2.5}$ over North America by individual models.

REFERENCES

Ackermann, I. J., Hass, H., Memmsheimer, M., Ebel, A., Binkowski, F. S., and Shankar, U.: Modal aerosol dynamics model for Europe: development and first applications, Atmos. Environ., 32, 2981–2999, doi:10.1016/S1352-2310(98)00006-5, 1998.

Ahmadov, R., McKeen, S. A., Robinson, A., Bahreini, R., Middlebrook, A., de Gouw, J., Meagher, J., Hsie, E., Edgerton, E., Shaw, S., and Trainer, M.: A volatility basis set model for summertime secondary organic aerosols over the eastern United States in 2006, J. Geophys. Res., 117, D06301, doi:10.1029/2011JD016831, 2012.

Appel, K. W., Pouliot, G. A., Simon, H., Sarwar, G., Pye, H. O. T., Napelenok, S. L., Akhtar, F., and Roselle, S. J.: Evaluation of dust and trace metal estimates from the Community Multiscale Air Quality (CMAQ) model version 5.0, Geosci. Model Dev., 6, 883–899, doi:10.5194/gmd-6-883-2013, 2013.

Banzhaf, S., M. Schaap, A. Kerschbaumer, E. Reimer, R. Stern, E. van der Swaluw, and Builtjes, P.: Implementation and evaluation of pH-dependent cloud chemistry and wet deposition in the chemical transport model REM-Calgrid, Atmos. Environ., 49, 378–390, doi:10.1016/j.atmosenv.2011.10.069, 2012.

Beltman, J. B., Hendriks, C., Tum, M., and Schaap, M.: The impact of large scale biomass production on ozone air pollution in Europe, Atmos. Environ., 71, 352–363, doi:10.1016/j.atmosenv.2013.02.019, 2013.

Bergström, R., Denier van der Gon, H. A. C., Prévôt, A. S. H., Yttri, K. E., and Simpson, D.: Modelling of organic aerosols over Europe (2002–2007) using a volatility basis set (VBS) framework: application of different assumptions regarding the formation of secondary organic aerosol, Atmos. Chem. Phys., 12, 8499–8527, doi:10.5194/acp-12-8499-2012, 2012.

Bieser, J., Aulinger, A., Matthias, V., Quante, M., and Builtjes, P.: SMOKE for Europe – adaptation, modification and evaluation of a comprehensive emission model for Europe, Geosci. Model Dev., 4, 47–68, doi:10.5194/gmd-4-47-2011, 2011a.

Bieser, J., Aulinger, A., Matthias, V., Quante, M., and Denier van der Gon, H. A. C.: Vertical emission profiles for Europe based on plume rise calculations, Environ. Pollut., 159, 2935–2946, doi:10.1016/j.envpol.2011.04.030, 2011b.

Brandt, J., Silver, J. D., Frohn, L. M., Geels, C., Gross, A., Hansen, A. B., Hansen, K. M., Hedegaard, G. B., Skjøth, C. A., Villadsen, H., Zare, A., and Christensen, J. H.: An integrated model study for Europe and North America using the Danish Eulerian Hemispheric Model with focus on intercontinental transport, Atmos. Environ., 53, 156–176, 2012.

Byun, D. W. and Schere, K.: Review of the governing equations, computational algorithms, and other components of the Models- 3 community Multiscale Air Quality (CMAQ) modeling system, Appl. Mech. Rev., 59, 51–77, 2006.

Damski, J., Thölix, L., Backman, L., Taalas, P., and Kulmala, M.: FinROSE: middle atmospheric chemistry transport model, Boreal Environ. Res. 12, 535–550, 2007.

de Leeuw, G., Neele, F. P., Hill, M., Smith, M. H., and Vignati, E.: Production of sea spray aerosol in the surf zone, J. Geophys. Res., 105, e29409, doi:10.1029/2000JD900549, 2000.

Doms, G.: A Description of the Nonhydrostatic Regional COSMO model, Part I: Dynamics and Numerics, Tech. rep., Deutscher Wetterdienst, available at: http://www.cosmo-model.org/content/model/documentation/core/cosmoDyncsNumcs.pdf.

Donahue, N. M., Robinson, A. L., Stanier, C. O., and Pandis, S. N.: Coupled partitioning, dilution, and chemical aging of semivolatile organics, Environ. Sci. Technol., 40.8, 635–2643, 2006.

Emberson, L. D., Ashmore, M. R., Simpson, D., Tuovinen, J.-P., and Cambridge, H. M.: Towards a model of ozone deposition and stomatal uptake over Europe. EMEP/MSC-W 6/2000, Norwegian Meteorological Institute, Oslo, Norway, 57 pp., 2000a.

Emberson, L. D., Ashmore, M. R., Simpson, D., Tuovinen, J.-P., and Cambridge, H. M.: Modelling stomatal ozone flux across Europe, Water Air Soil Pollut., 109, 403–413, 2000b.

Fast, J. D., Gustafson Jr., W. I., Easter, R. C., Zaveri, R. A., Barnard, J. C., Chapman, E. G., Grell, G. A., and Peckham, S. E.: Evolution of ozone, particulates, and aerosol direct radiative forcing in the vicinity of Houston using a fully coupled meteorology-chemistry-aerosol model, J. Geophys. Res., 111, D21305, doi:10.1029/2005JD006721, 2006.

Foley, K. M., Roselle, S. J., Appel, K. W., Bhave, P. V., Pleim, J. E., Otte, T. L., Mathur, R., Sarwar, G., Young, J. O., Gilliam, R. C., Nolte, C. G., Kelly, J. T., Gilliland, A. B., and Bash, J. O.: Incremental testing of the Community Multiscale Air Quality (CMAQ) modeling system version 4.7, Geosci. Model Dev., 3, 205–226, doi:10.5194/gmd-3-205-2010, 2010.

Fountoukis, C. and Nenes, A.: ISORROPIA II: a computationally efficient thermodynamic equilibrium model for KC–Ca2C–Mg2C–NHC4 –NaC–SO2□4 –NO□3 –Cl□–H2O aerosols, Atmos. Chem. Phys., 7, 4639–4659, doi:10.5194/acp-7-4639-2007, 2007.

Gantt, B., Kelly, J. T., and Bash, J. O.: Updating sea spray aerosol emissions in the Community Multiscale Air Quality (CMAQ) model version 5.0.2, Geosci. Model Dev., 8, 3733–3746, doi:10.5194/gmd-8-3733-2015, 2015.

Geyer, B.: High-resolution atmospheric reconstruction for Europe 1948–2012: coastDat2, Earth Syst. Sci. Data, 6, 147–164, doi:10.5194/essd-6-147-2014, 2014.

Gilliam, R. C., Godowitch, J. M., and Rao, S. T.: Improving the horizontal transport in the lower troposphere with four dimensional data assimilation, Atmos. Environ., 53, 186–201, doi:10.1016/j.atmosenv.2011.10.064, 2012.

Ginoux, P., Chin, M., Tegen, I., Prospero, J. M., Holben, B., Dubovik, O., and Lin, S. J.: Sources and distributions of dust aerosols simulated with the GOCART model, J. Geophys. Res., 106, 20255–20273, 2001.

Gong, S. L.: A parameterization of sea-salt aerosol source function for sub- and super-micron particles, Global Biogeochem. Cy., 17, 1097–1104, 2003.

Grell, G. A. and Devenyi, D.: A generalized approach to parameterizing convection combining ensemble and data assimilation techniques, Geophys. Res. Lett., 29, 1693, doi:10.1029/2002GL015311, 2002.

Grell, G. A. and Freitas, S. R.: A scale and aerosol aware stochastic convective parameterization for weather and air quality modeling, Atmos. Chem. Phys., 14, 5233–5250, doi:10.5194/acp-14-5233-2014, 2014.

Guenther, A., Karl, T., Harley, P., Wiedinmyer, C., Palmer, P. I., and Geron, C.: Estimates of global terrestrial isoprene emissions using MEGAN (Model of Emissions of Gases and Aerosols from Nature), Atmos. Chem. Phys., 6, 3181–3210, doi:10.5194/acp-6-3181-2006, 2006.

Guenther, A. B., Jiang, X., Heald, C. L., Sakulyanontvittaya, T., Duhl, T., Emmons, L. K., andWang, X.: The Model of Emissions of Gases and Aerosols from Nature version 2.1 (MEGAN2.1): an extended and updated framework for modeling biogenic emissions, Geosci. Model Dev., 5, 1471–1492, doi:10.5194/gmd-5-1471-2012, 2012.

Hogrefe, C., Pouliot, G., Wong, D., Torian, A., Roselle, S., Pleim, J., and Mathur, R.: Annual application and evaluation of the online coupled WRF-CMAQ system over North America under AQMEII phase 2, Atmos. Environ., 115, 683–694, 2015.

Hong, S.-Y., Noh, Y., and Dudhia, J.: A new vertical diffusion package with explicit treatment of entrainment processes, Mon. Weather Rev., 134, 2318–2341, 2006.

Kalnay, E., Kanamitsu, M., Kistler, R., Collins, W., Deaven, D., Gandin, L., Iredell, M., Saha, S., White, G., Woollen, J.,Zhu, Y., Chelliah, M., Ebisuzaki, W., Higgins, W., Janowiak, J., Mo, K. C., Ropelewski, C., Wang, J., Leetmaa, A.,Reynolds, R., Jenne, R., and Joseph, D.: The NCEP/NCAR 40-year reanalysis project, B. Am. Meteorol. Soc., 77, 437–471, 1996.

Kim, S. W., Heckel, A., Frost, G. J., Richter, A., Gleason, J., Burrows, J. P., McKeen, S., Hsie, E. Y., Granier, C., and Trainer, M.: NO2 columns in the western United States observed from space and simulated by a regional chemistry model and their implications for NOx emissions, J. Geophys. Res.-Atmos., 114, D11301, doi:10.1029/2008jd011343, 2009.

Lattuati M.: Impact des emissions européennes sur le bilan d’ozone troposphérique à l’interface de l’Europe et de l’Atlantique Nord: apport de la modélisation lagrangienne et des mesures en altitude, PhD Thesis, Université Pierre et Marie Curie, Paris, France, 1997.

Lin, Y. L., Farley, R. D., and Orville, H. D.: Bulk parameterization of the snow field in a cloud model, J. Clim. Appl. Meteorol., 22, 1065–1092, 1993.

Martensson, E. M., Nilsson, E. D., de Leeuw, G., Cohen, L. H., and Hansson, H.-C.: Laboratory simulations and parameterization of the primary marine aerosol production, J. Geophys. Res.-Atmos., 108, AAC 15-1–AAC 15-12, doi:10.1029/2002JD002263, 2003.

Monahan, E. C., Spiel, D. E., and Davidson, K. L.: A model of marine aerosol generation via whitecaps and wave disruption, in: Oceanic Whitecaps and their role in air/sea exchange, edited by: Monahan, E. C. and Mac Niocaill, G., Reidel, Norwell, Mass., USA, 167–174, 1986.

Nenes, A, Pilinis, C., and Pandis, S. N.: Continued Development and Testing of a New Thermodynamic Aerosol Module for Urban and Regional Air Quality Models, Atmos. Environ., 33, 1553–1560, 1999.

Otte, T. L. and Pleim, J. E.: The Meteorology-Chemistry Interface Processor (MCIP) for the CMAQ modeling system: updates through MCIPv3.4.1, Geosci. Model Dev., 3, 243–256, doi:10.5194/gmd-3-243-2010, 2010.

Otte, T. L., Pouliot, G., Pleim, J. E., Young, J. O., Schere, K. L., Wong, D. C., Lee, P. C. S., Tsidulko, M., McQueen, J. T., Davidson, P., Mathur, R., Chuang, H. Y., DiMego, G., and Seaman, N. L.: Linking the Eta model with the Community Multiscale Air Quality (CMAQ) modeling system to build a national weather forecasting system, Weather Forecast., 20, 367–384 2005.

Pleim, J. E. and Xiu, A.: Development of a land surface model, Part II: data assimilation, J. Appl. Meteorol., 42, 1811–1822, 2003.

Pleim, J. E. and Gilliam, R.: An indirect data assimilation scheme for deep soil temperature in the Pleim-Xiu land surface model, J. Appl. Meteorol. Clim., 48, 1362–1376, 2009.

Poppe, D., Andersson-Sköld, Y., Baart, A., Builtjes, P. J. H., Das, M., Fiedler, F., Hov, O., Korchner, F., Kuhn, M., Makar, P. A., Milford, J. B., Roemer, M. G. M., Runhke, R., Simpson, D., and Stockwell, W. R.: Gas-Phase reactions in atmospheric chemistry and transport models, Tech Rep. Garmisch-Partenkirchen, Germany: Eurotrac report, 1996.

Poupkou, A., Giannaros, T., Markakis, K., Kioutsioukis, I., Curci, G., Melas, D., and Zerefos, C.: A model for European Biogenic Volatile Organic Compound emissions: Software development and first validation, Environ. Model. Softw., 25, 1845–1856, doi:10.1016/j.envsoft.2010.05.004, 2010.

Ritter, B. and Geleyn, J. F.: A comprehensive radiation scheme for numerical weather prediction models withpotential applications in climate simulations, Mon. Weather Rev., 120, 303–325, doi:10.1175/1520-0493(1992)120<0303:ACRSFN>2.0.CO;2, 1992.

Sarwar, G., Appel, K. W., Carlton, A. G., Mathur, R., Schere, K., Zhang, R., and Majeed, M. A.: Impact of a new condensed toluene mechanism on air quality model predictions in the US, Geosci. Model Dev., 4, 183–193, doi:10.5194/gmd-4-183-2011, 2011.

Sauter, F., Swaluw, E. van der, Manders-Groot, A., Wichink Kruit, R., Segers, A., and Eskes, H.: LOTOS-EUROS v1.8 Reference Guide, TNO Report TNO-060-UT-2012-01451, Utrecht, the Netherlands, 2012.

Schaap, M., Sauter, F., Timmermans, R. M. A., Roemer, M., Velders, G., Beck, J., and Builtjes, P.: The LOTOS-EUROS model: description, validation and latest developments, Int. J. Environ. Pollut., 32, 270–290, 2008.

Schaap, M., Manders, A., Hendriks, E., Cnossen, J., Segers, A., Denier van der Gon, H., Jozwicka, M., Sauter, F., Velder, G., Matthijsen, J., and Builtjes, P.: Regional Modelling of Particulate Matter for the Netherlands, Technical background report BOP, report 500099008, Netherlands Environmental Assessment Agency, 2009.

Schaettler, U., Doms, G., and Schraff, C.: A Description of the Nonhydrostatic Regional COSMO-Model Part VII: User's Guide, Tech. rep., Deutscher Wetterdienst, 2008.

Schrodin, R. and Heise, E.: The multi-layer-version of the DWD soil model TERRA/LM,Tech. Rep., No. 2, Consortium for Small-Scale Modelling (COSMO), available at: http://www2.cosmo-model.org/content/model/documentation/techReports/docs/techReport02.pdf, 2001.

Seifert, A. and Beheng, K. D.: A double-moment parameterization for simulating autoconversion, accretion and selfcollection, Atmos. Res., 59–60, 265–281, doi:10.1016/S0169-8095(01)00126-0, 2001.

Shrivastava, M., Fast, J., Easter, R., Gustafson Jr., W. I., Zaveri, R. A., Jimenez, J. L., Saide, P., and Hodzic, A.: Modeling organic aerosols in a megacity: comparison of simple and complex representations of the volatility basis set approach, Atmos. Chem. Phys., 11, 6639–6662, doi:10.5194/acp-11-6639-2011, 2011.

Skamarock, W. C., Klemp, J. B., Dudhia, J., Gill, D. O., Barker, D. M., Duda, M. G., Huang, X.-Y., Wang, W., and Powers, J. G.: A description of the Advanced Research WRF version 3, National Center for Atmospheric Research Tech. Note, NCAR/TN-475CSTR, 113 pp., 2008.

Soares, J., Sofiev, M., and Hakkarainen, J.: Uncertainties of wildland fires emission in AQMEII phase 2 case study, Atmos. Environ., 115, 361–370, doi:10.1016/j.atmosenv.2015.01.068, 2015.

Sofiev, M.: A model for the evaluation of long-term airborne pollution transport at regional and continental scales, Atmos. Environ., 34, 2481–2493, doi:10.1016/S1352-2310(99)00415-X, 2000.

Sofiev, M., Soares, J., Prank, M., de Leeuw, G., and Kukkonen, J.: A regional-to-global model of emission and transport of sea salt particles in the atmosphere, J. Geophys. Res., 116, D21302, doi:10.1029/2010JD014713, 2011.

Sofiev, M., Vira, J., Kouznetsov, R., Prank, M., Soares, J., and Genikhovich, E.: Construction of the SILAM Eulerian atmospheric dispersion model based on the advection algorithm of Michael Galperin, Geosci. Model Dev., 8, 3497–3522, doi:10.5194/gmd-8-3497-2015, 2015.

Stockwell, W. R., Kirchner, F. K., Kuhn, M., and Seefeld, S.: A new mechanism for regional atmospheric chemistry modeling, J. Geophys. Res., 102, 25847–25879, doi:10.1029/97JD00849, 1997.

Stockwell,W. R., Middleton, P., Chang, J. S., and Tang, X.: The second generation regional acid deposition model chemical mechanism for regional air quality modeling, J. Geophys. Res. 95, 16343e16367, doi:10.1029/JD095iD10p16343, 1990.

Tiedtke, M.: A comprehensive mass flux scheme for cumulus parameterization in large-scale models, Mon. Weather Rev., 117, 1779–1800, 1989.

Tuccella, P., Curci, G., Grell, G. A., Visconti, G., Crumeyrolle, S., Schwarzenboeck, A., and Mensah, A. A.: A new chemistry option in WRF-Chem v. 3.4 for the simulation of direct and indirect aerosol effects using VBS: evaluation against IMPACT-EUCAARI data, Geosci. Model Dev., 8, 2749–2776, doi:10.5194/gmd-8-2749-2015, 2015.

Walcek, C. J. and Taylor, G. R.: A theoretical method for computing vertical distributions of acidity and sulfate production within cumulus clouds, J. Atmos Sci., 43, 339–355, doi:0.1175/1520-0469(1986)043< 0339:ATMFCV>2.0.CO;2, 1986.

Whitten, G. Z., Heo, G., Kimura, Y., McDonald-Buller, E., Allen, D. T., Carter, W. P. L., and Yarwood, G.: A new condensed toluene mechanism for Carbon Bond: CB05-TU, Atmos. Environ., 44, 5346–5355, 2010.

Yarwood, G., Rao, S., Yocke, M., and Whitten, G.: Updates to the Carbon Bond Chemical Mechanism: CB05, Final Report to the US EPA, RT-0400675, available at: http://www.camx.com/files/cb05_final_report_120805.aspx, 2005.

Zare, A., Christensen, J. H., Gross, A., Irannejad, P., Glasius, M., and Brandt, J.: Quantifying the contributions of natural emissions to ozone and total fine PM concentrations in the Northern Hemisphere, Atmos. Chem. Phys., 14, 2735–2756, doi:10.5194/acp-14-2735-2014, 2014.
